# Diatoms-endoparasite association in fish from the marine pacific coast of Colombia (Buenaventura)

**DOI:** 10.1371/journal.pone.0312015

**Published:** 2024-12-27

**Authors:** Vanessa Potosi-Pai, Carlos E. Agudelo Morales, Javier Antonio Benavides-Montaño

**Affiliations:** 1 Animal Science Department, Universidad Nacional de Colombia, Palmira Valle, Colombia; 2 Biology Department, Microscopy and Imagen Lab, Universidad Nacional de Colombia, Palmira Valle, Colombia; 3 Parasitology Immunology and Infectious Disease Lab, Universidad Nacional de Colombia, Palmira Valle, Colombia; Beni Suef University Faculty of Veterinary Medicine, EGYPT

## Abstract

The association of parasites and diatoms has been previously reported as an important mechanism to control bacteria and parasites to avoid resistance to chemical usage. The aim of this study was to investigate the association between diatoms genus and parasites within the gastrointestinal compartments (GICs) of commercial fish in fisheries of the marine Pacific coast of Colombia (Buenaventura). A total of 104 GICs from marine fish were sampled. The GICs analysis revealed 14 diatom genera (N = 14). The most prevalent were *Coscinodiscus* spp., which was present in 58/104 samples, 55.8% [95% CI = 37.5–62.1%]; *Cyclotella* spp., 28/104, 26.9% [95% CI = 0–25%]; *Paralia* spp., 26/104, 25% [95% CI = 12.5–44.8%]; *Gyrosigma* spp., 11/104, 10.6% [95% CI = 0–33.3%]; *Navicula* spp., 11/104, 10.6%, [95% CI = 0–20.7%]. The GICs analysis revealed a diversity of genera parasites. The most prevalent were Ameboid cysts, 25/104, 24% [95% CI = 12.5–48.3%]; *Eimeria* spp., 11/104, 10.6% [95% CI = 10.3–15.7%]; *Anisakis* spp., 29/104, 27.1% [95% CI = 27.1 (SD±12.9%)]. This is the first report concerning diatoms and parasites association in fish from the Pacific Coast of Colombia and highlights the relevance of *Coscinodiscus* spp. and *Gyrosigma* spp. as important diatoms and potential candidates for studying pharmaceutical action in aquaculture. Further studies about diatoms-parasites association in aquaculture are required.

## 1. Introduction

Diatoms are unicellular microscopic algae that belong to the class Bacillariophyta. With over 100,000 known species, are a major group of algae, specifically a type of phytoplankton, characterized by their unique silica cell walls, known as frustules [1, 2]. These microscopic, photosynthetic organisms play a crucial role in aquatic ecosystems, contributing significantly to the primary production of oxygen and forming the base of the food chain in marine and freshwater environments [1, 2]. These phytoplankton are ubiquitous in aquatic environments, whether marine or freshwater, and serve as significant biomarkers [2–4]. Some species exhibit pharmaceutical properties due to their diverse components, which have been reported to control bacteria and parasites. Additionally, they are valuable as a source of essential fatty acids like Eicosapentaenoic Acid (EPA) and Docosahexaenoic Acid (DHA), as well as various bioactive molecules, making them relevant to the nutraceutical industry [1, 5–9].

Marine fish parasites encompass a wide array of species, comprising both protozoan and metazoan parasites [10, 11]. These parasites present significant economic burdens, with costs related to parasitic infections in finfish aquaculture, reaching up to 10% of the projected fish yield worldwide [12], but also, they represent a threat to both marine and freshwater fish, exacerbating challenges such as habitat loss, pollution, invasive species, and overfishing. The emergence of these parasites has been further fueled by global warming [5, 13–15]. In South America, and specifically Colombia, various parasites have been documented to impact human health, with clinical cases, as a consequence of global tourism and consuming exotic foods. Individuals can inadvertently become infected by sampling ethnic dishes, traditional cuisine, or unique culinary preparations [16–19].

The association study between parasites and diatoms has shown promise as an innovative approach to controlling parasitic infections in fish, particularly in aquaculture, where resistance to traditional treatments is a growing concern [5, 8, 20–23]. Despite extensive research, there is a significant knowledge gap regarding these associations in the tropical ecosystems of Colombia’s Pacific coast [1]. This region possessses significantive species richness, especially within its aquatic ecosystems, where diatoms from the Bacillariophyta order thrive.

Colombia is a unique tropical country in South America, having coasts on both the tropical Pacific Ocean and the Caribbean Sea with a total shoreline of nearly 3,000 km, is recognized by its rich biodiversity within its aquatic ecosystems, including a variety of microorganisms such as algae, particularly diatoms from the Bacillariophyta order [2–4, 24].

In order to contribute to the discussion regarding how fish manage endoparasites disease, and considering that there is minimal study on this field [25], we hypothesize that diatoms are important elements for fish diet and plays a significant role, probably to treat or minimize the endoparasite charges. The main objective of this research was to investigate the association between diatoms genus and parasites within the gastrointestinal compartments (GICs) of commercial fish in fisheries of the marine pacific coast of Colombia (Buenaventura). This aimed to better understand the zoo pharmacognosy in commercial fish, the self-medication behavior of fishes in this region, as well as to analyze the structure and vulnerability of the host-parasite networks field using diatoms as biomarkers [26, 27].

## 2. Materials and methods

### 2.1 Ethical approval for the study

The research described in this study did not involve experimentation on animals. Samples were obtained following the regulations governing fisheries markets and community practices in Buenaventura, Colombia [28, 29]. Specifically, the sampling was conducted at the Puente del Piñal, San Antonio ‐ Buenaventura, with full adherence to local guidelines and community consent [30]. All aspects of this research adhered to the highest ethical standards and respected the welfare of animals.

### 2.2 Description of the study area

**Fig 1:**
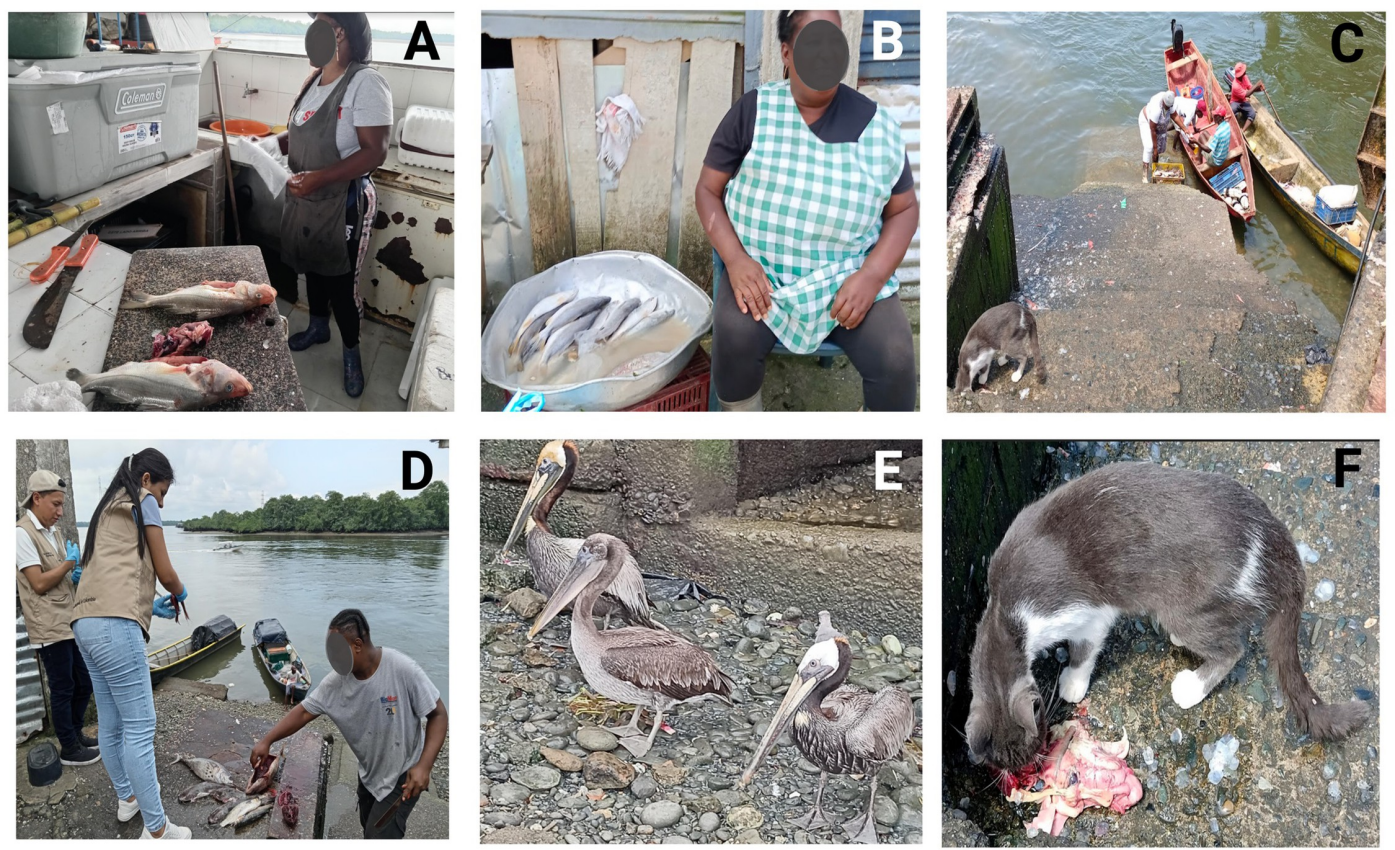
Samples were collected from Puente el Piñal, and San Antonio. Ranging in elevation from 0 to 7 m.a.s.l. (A, B) Platonera in Puente el Piñal, preparing and selling market fish products; (C) fishing boat arriving after fishing at Puente el Piñal; (D) fishermen, eviscerating fish; (E) pelicans in the Puente el Piñal; (F), cat eating gastrointestinal compartments of fish. Created in BioRender. Benavides, J. (2024) BioRender.com/e10g125.

We sampled in the Piñal Bridge in Buenaventura in the Department of Valle del Cauca, Colombia, following these parameters: first, it is the one of the main fisheries centers that distribute and merchandise the product to inhabitants, and supplies restaurants and markets in Buenaventura; secondly, it is a significant species richness center, with cultural practices and traditional sanitary regulation. Thirdly, the Buenaventura harbor is one of the leading international markets in Colombia [31, 32]. Buenaventura is in the estuary of Buenaventura Bay, at the Tropical Eastern Pacific (3° 52′ 59,7” N to 77° 3´ 20”). The estuary spans approximately 70 km^2^ and has a 16-km-long and 5-m-deep central canal field (Fig 1) [33].

### 2.3 Type of study

This cross-sectional study sought to assess the associations between diseases or health disturbances and other variables of interest in a specific population and time. The presence or absence of the parasitic infection and its variables was examined in a sample without considering the temporal sequence of cause and effect [34]. Prevalence (proportion infected), mean intensity (parasites per infected individual), and mean abundance (parasites per individuals examined) were determined according to methods used by Bush et al. [35]. The prevalence of gastrointestinal parasites was estimated using prevalence (p) = the number of total cases divided by the sum of the population at the moment (×100). The data was expressed in percentages (%). This research employed three stool samples to accurately diagnose intestinal parasitic infections (IPI), with a 95% confidence interval (CI). T-test and Mann-Whitney or Wilcoxon signed rank test were employed to known significance assuming normal distribution employing GraphPad Prism 10. The value for the significance of the association and allowable error was 0.05 [34]. Parasite prevalence was calculated according to Margolis et al. (1982) [36] and Bush et al. (1997) [37, 38]. Preliminary parasite identification was based on the keys of Anderson (2000) [39] and Reichenbach-Klinke, H. 1. (1975) [40, 41]. A retrospective study cohort of risk factor in common (Exposed subjects, Non-exposed subjects) let us identify the Odds ratio (OR) association of GICs exposure and not exposure to diatoms and parasites. ’Exposed’ is designated as (E) and Non-exposed’ as (U); therefore = E/ U [7, 42–44].

### 2.4 Fish sample processing

In this study, 104 gastrointestinal digestive tract samples were collected from asymptomatic fish belonging to 22 different fish species on three sampling dates: March 19, 2023 (24 compartments), October 7, 2023 (29 compartments), and March 9, 2024 (51 compartments). The samples were gathered at two different points at 10 km from each other. The gastrointestinal compartments of fish were stored in a propylene bag. The gastrointestinal samples were placed in centrifuge tubes (50 ml) with 10% formalin and other samples in methanol for future DNA studies. They were then transported on ice to the Parasitology, Immunology, and Infectious Diseases Laboratory of the National University of Colombia ‐ Faculty of Agricultural Sciences, Palmira Campus (Palmira, Valle del Cauca, Colombia). Endoparasites were stored in 1.5 ml tubes in 70% alcohol and methanol [31]. The identification was based on previously published keys and morphological features, as suggested in the literature field [10, 41, 45]. The gastrointestinal tracts were collected from diverse species of available fish in each sampling time: *Anisotremus* spp (Curruco), *Bagre panamensis* (Canchimala), *Bagre pinnimaculatus* (Barbinche), *Caranx caninus* (Jurel rayado), *Caranx sexfasciatus* (Burique, Jurel ojón), *Caranx vinctus* (7 presas), *Centropomus unionensis*(Gualajo), *Cynoscion albus* (Pelada), *Cynoscion praedatorius* (Boco), *Cynoscion squamipinnis* (Corvina), *Eugerres periche* (Carpia), *Lutjanus colorado* (Pargo rojo), *Lutjanus guttatus* (Pargo colorado), *Menticirrhus panamensis* (Botellona), *Mugil curema* (Lisa), *Nematistius pectoralis* (Pejegallo), *Notarius armbrusteri* (Ñato), *Oreochromis niloticus* (Mojarra), *Parapsettus panamensis* (Palma), *Peprilus snyderi* (Manteco), *Strongylura fluviatilis* (Aguja) and *Thunnus alalunga* (Atún) [46, 47]. The parasite analysis was performed by bright field and contrast phase microscopy, using a ZEISS ZXIO microscope, AxioCam ERc5s ICc 1 and using flotation with the Sheather technique, and sedimentation.

### 2.5 Dissection and parasite examination

For endoparasites, the entire alimentary tract was removed from the body, placed in vertebrate saline (10% seawater and 90% freshwater; one part seawater to four parts freshwater), split with a lengthwise incision, and its internal surface and contents were inspected. Gut wash techniques were performed under Cribb and Bray’s (2010) [48]. The gut cavity and all internal organs were superficially inspected for the presence of parasites. Helminth parasites were fixed in near-boiling saline and preserved in 10% formalin. All other parasites were preserved in methanol. Fish samples were taken during regular sale activities in the Puente El Piñal. The fishes offered for sale were dissected, and their compartments were stored and preserved in the field; carcasses were not examined [49]. Fecal samples were collected, filtered, and stored in a 50 ml tube and centrifugated at 2200 rpm for 5 minutes. Supernatants were eliminated by washing the pellet three times. Samples were analyzed by microscopical techniques (sedimentation and flotation) and examined at 40X in order to identify diatoms, parasite cysts, and eggs [34, 50]. The L4 adult larva parasites found in the gastrointestinal tract of infected fish were fixed in methanol. The nematodes were dehydrated in a successive solution of alcohols (30%, 50%, 70%, and 100% alcohol) and allowed to dry in order to be observed in a confocal microscope. Nematodes were fixed with coal tape and exposed to 80 seconds cover with gold particles, 10–15 nm thick. Afterwards, they were covered with gold and observed in a scanning JCM 5000 NeoScope electron microscope (SEM). Samples were transferred to the microscope in order to be visualized at 10 kV.

## 3. Results

**Fig 2:**
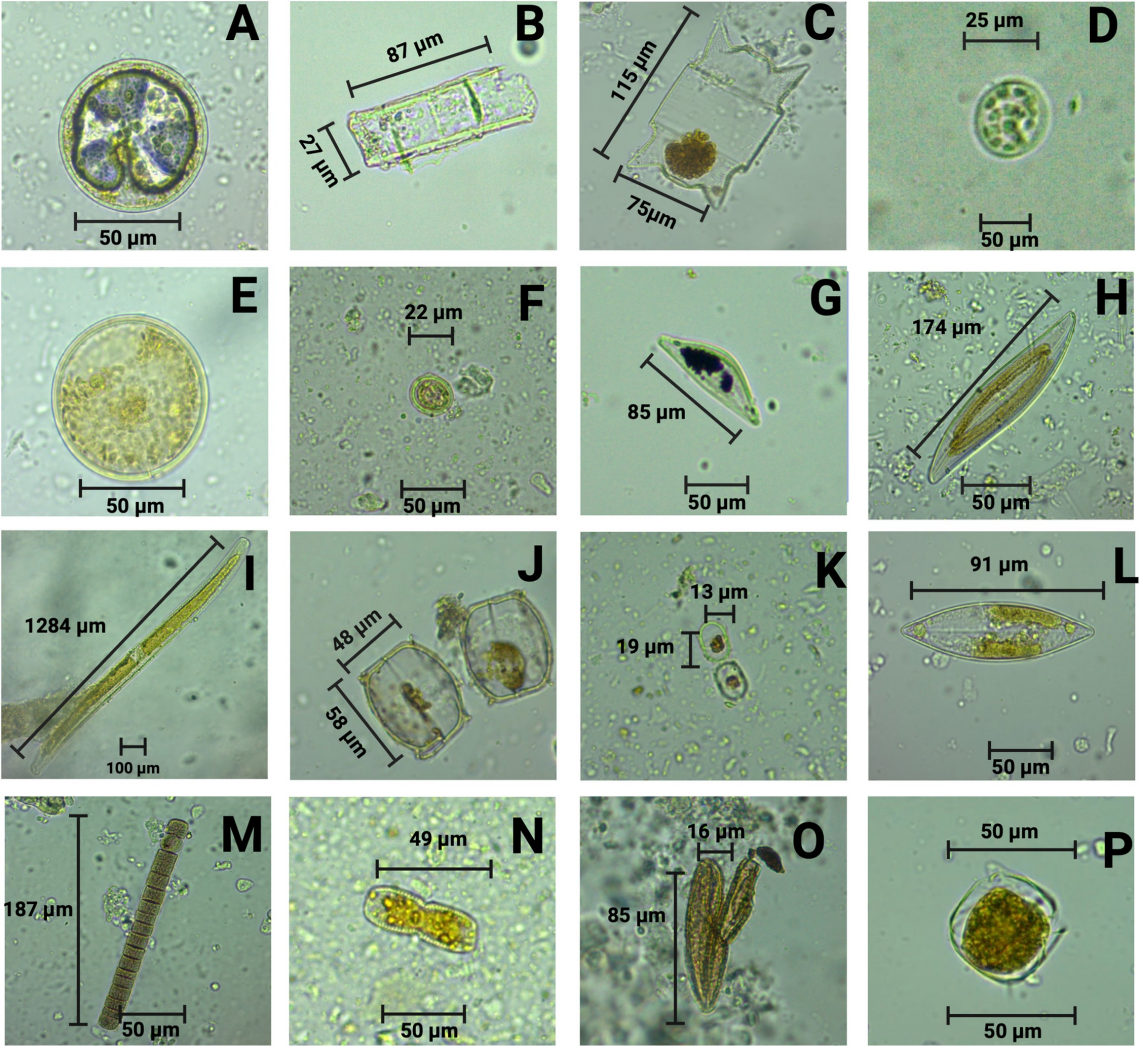
Species of diatoms present in marine fish intestine from the pacific of Colombia. A) *Actinoptychus* spp.; B) *Aulacoseira* spp.; C) *Biddulphia* spp; D) *Botrydiopsis* spp; E) *Coscinodiscus* spp.; F) *Cyclotella* spp; G) *Cymbella* spp.; H, I) *Gyrosigma* spp.; J, K) *Melosira* spp.; L) *Navicula* spp.; M) *Paralia* spp.; N) *Skeletonema* spp.; O) *Torodinium* spp.; P) *Unruhdinium* spp. Created in BioRender. Created in BioRender. Benavides, J. (2024) BioRender.com/z47t234.

From 104 gastrointestinal compartments acquired from marine fish, this study identified different species of diatoms and parasites. 14 genus of diatoms were found: *Actinoptychus* spp., *Aulacoseira* spp., *Biddulphia* spp., *Botrydiopsis* spp., *Cyclotella* spp., *Cymbella* spp., *Coscinodiscus* spp., *Gyrosigma* spp., *Melosira* spp., *Navicula* spp., *Paralia* spp., *Skeletonema* spp., *Torodinium* spp., and *Unruhdinium* spp. *Coscinodiscus* emerged as the most prevalent diatom, appearing in 58/104 analyzed compartments. This diatom was present in eight species, and it was most frequently identified in *Mugil curema* in all sampling (Fig 2, Table 1, S1 Fig and S1 Table). *Cyclotella* spp. also had a high prevalence, 28/104 (26.9%) in *Anisotremus* spp. and in the last sampling. In the case of *Paralia* spp., it was found in 26/104, 25%; *Centropomus unionensis* and *Mugil* curema were identified in all samples. *Gyrosigma* spp. at 11/104, 10.6%; was present in three species *Mugil curema*, *Oreochromis niloticus*, *Parapsettus panamensis* in two sampling times. *Navicula* spp. at 11/104, 10.6%, (Fig 2, Table 1, S1 Fig and S1 Table.)

**Table 1:** Diatoms found in the gastrointestinal compartments of fish population in Buenaventura port of Colombia.

Diatoms	Fish species	Total no. individuals	Prevalence means %	Prevalence IC 95%
*Actinoptychus* spp.	*Bagre pinnimaculatus*, *Anisostremus* spp.	13	6.5 (SD±5.3)	0–12
*Aulacoseira* spp.	*Eugerres periche*	11	10.6 (SD ± 9.3)	0–20.7
*Biddulphia* spp.	*Centropomus unionensis*, *Bagre pinnimaculatus*, M*ugil curema*	3	2.9 (SD±6.5)	0–12.5
*Botrydiopsis* spp.	*Bagre panamensis*, *Mugil curema*	11	10.6 (SD±10.7)	0–20.7
*Cyclotella* spp.	*Anisotremus*spp	28	26.9 (SD±14.8)	0–25.4
*Cymbella* spp.	*Parapsettus panamensis*	2	1.9 (SD±4.3)	0–8.3
*Coscinodiscus* spp.	*Mugil curema*,	58	55.8 (SD±12.4)	37.5–62.1
	*Bagre pinnimaculatus*			
	*Oreochromis niloticus niloticus*			
	*Parapsettus panamensis*,			
	*Centropomus unionensis*			
	*Cynoscion albus*			
	*Eugerres periche*			
	*Notarius armbrusteri*			
*Gyrosigma* spp.	*Mugil curema*	11	10.6 (SD±15.3)	0–33.3
	*Oreochromis niloticus niloticus*			
	*Parapsettus panamensis*			
*Melosira* spp.	*Bagre panamensis*	5	4.8 (SD±4.9)	0–8.3
	*Mugil curema*			
*Navicula* spp.	*Centropomus unionensis*	11	10.6 (SD±9.8)	0–16.6
	*Eugerres periche*			
	*Mugil curema*			
*Paralia* spp.	*Centropomus unionensis*	26	25.0 (SD±14.9)	12.5–44.8
	*Mugil curema*			
*Skeletonema* spp.	*Mugil curema*	3	2.9 (SD±5.3)	0–10.3
*Torodinium* spp.	*Lutjanus guttatus*	3	2.9 (SD±2.2)	0–4.1
*Unruhdinium* spp.	*Mugil curema*	3	2.9 (SD±6.5)	0–12.5

**Fig 3:**
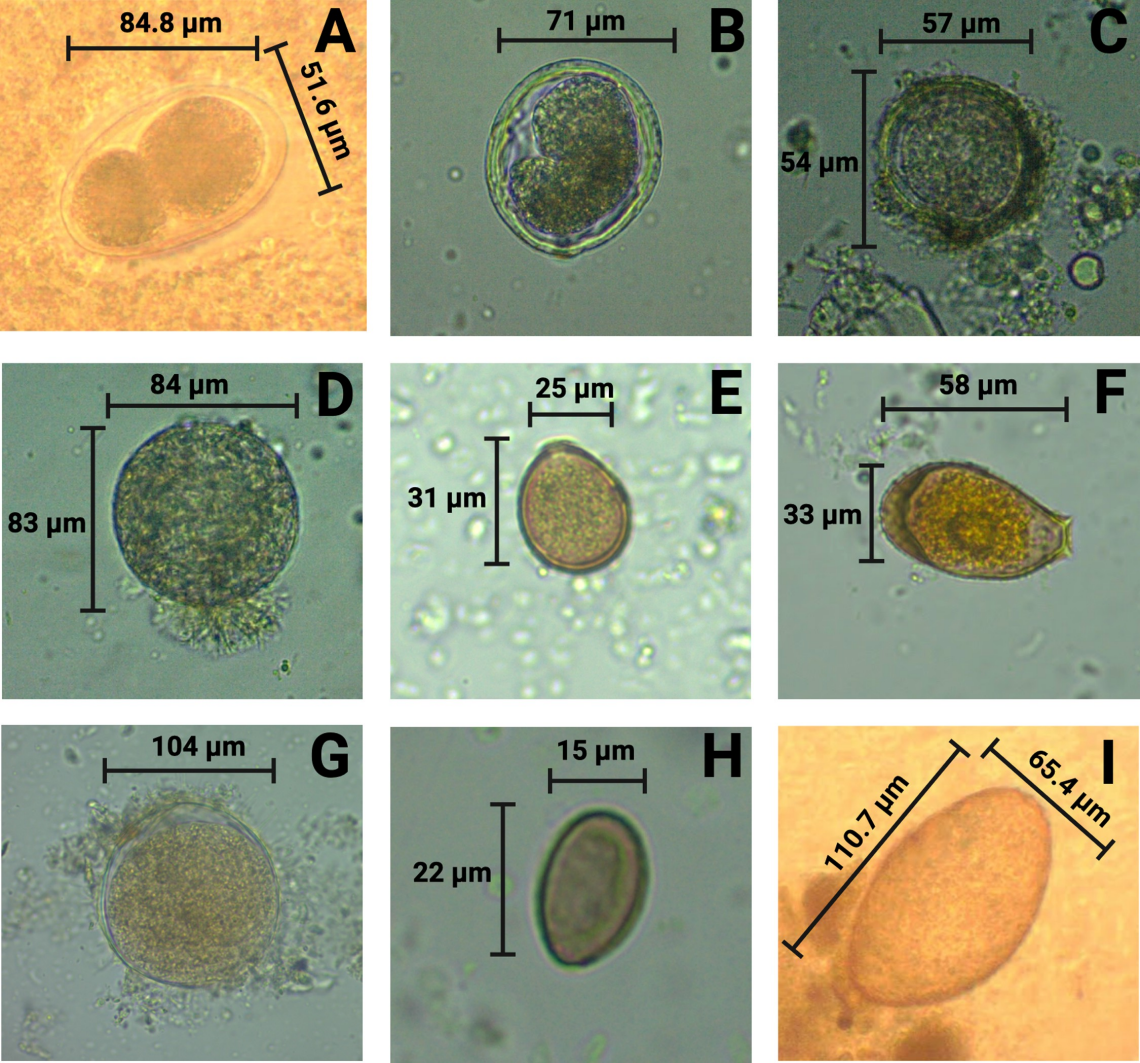
Parasites found from various Pacific fish species: *Anisakidae eggs* (A); *Contracaecum* spp. (B), *Anisakis* spp. (C), Cyst of a cilliated from *Cynoscion albus* (D), *Eimeria* spp. (E, F), *Macrostomorpha* spp. (G), *Metagonimus* spp. (H), *Paragonimus* spp. (I). *Created in BioRender*. *Benavides*, *J*. *(2024) BioRender*.*com/u24c141*.

**Fig 4:**
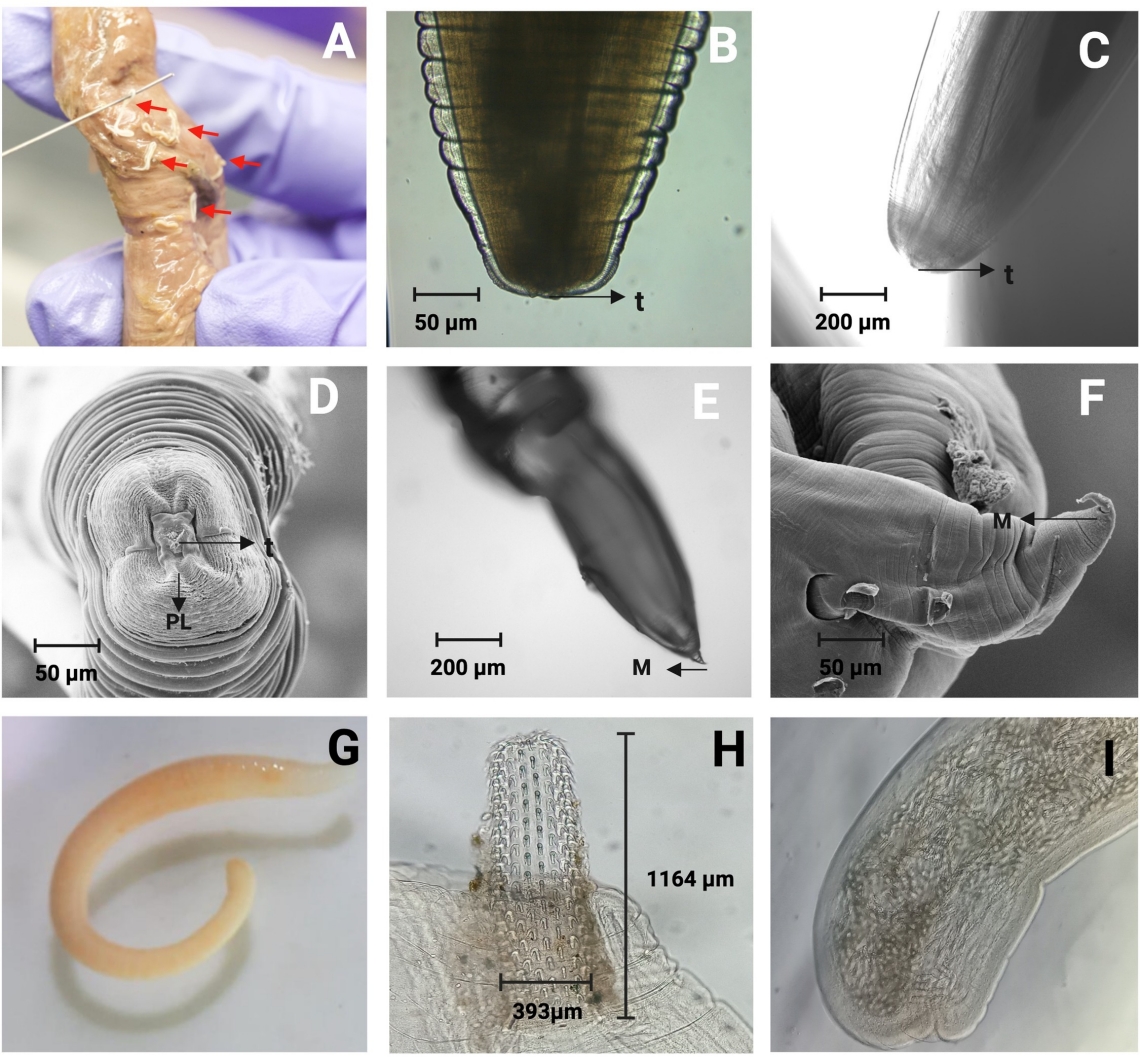
Helminths from *Mugil curema*, *Thunnus alalunga* and *Notarious armbrusteri*. Nematodes located on cerose gastric (A), Anisakidae family teeth observed 40X t: (B) Confocal, lateral view (C), *Anisakis* simplex *sensu lato*. anterior region (D), posterior or tail observed by confocal microscopy (E), SEM tail (F), *Acanthocephalus* spp. (G), *Acanthocephalus* spp. head with hooks (H), *Acanthocephalus* spp. tail (I). *Created in BioRender*. *Benavides*, *J*. *(2024) BioRender*.*com/h00l150*.

Endoparasites associated with diatoms, specifically *Anisakis* spp. and *Contracaecum* spp., were detected in the intestines of *Mugil curema* (commonly known as Lisa) at a prevalence ranging from 3.9% to 12.5%. *Mugil curema*, *Anisakis* spp. and *Contracaecum* spp. exhibited associations with various diatom species, including *Botrydiopsis* spp., *Coscinodiscus* spp., *Gyrosigma* spp., *Navicula* spp., *Paralia* spp., and *Unruhdinium* spp. Notably, during the third sampling, the species of diatoms decreased to a single species, *Coscinodiscus* spp., at a prevalence of 3.9%, while *Anisakis* spp. and *Contracaecum* spp. persisted (Figs 3 and 4 and Table 2, S2 Fig).

**Table 2:** Endoparasites found in the gastrointestinal compartments of fish population in Buenaventura port of Colombia.

EndoParasites	Fish species	Total no. individuals	Prevalence means %	Prevalence IC 95%
*Contracaecum* spp.	*Mugil curema*	10	9.6 (SD±2.1)	7.8–12.5
	*Thunnus alalunga*			
*Acanthocephalus* spp.	*Menticirrhus panamensis*	9	8.7 (SD±10.5)	4.2–15.7
	*Notarius armbrusteri*			
Ameboid cyst	*Notarius armbrusteri*, *Oreochromis niloticus niloticus*	25	24.0 (SD±16.2)	12.5–48.3
*Anisakis* spp.	*Mugil curema*	29	12.9 (SD±14.4)	27.1 ± 2.479
	*Cynoscion albus*			
	*Thunnus alalunga*			
	*Bagre pinnimaculatus*			
	*Anisotremus* spp.			
	*Cynoscion albus*			
*Cilliophora type Balantidium* spp.	*Cynoscion albus*	7	6.7 (SD±4.8)	0–10.3
*Eimeria* spp.	*Notarius armbrusteri*	11	10.6 (SD±2.4)	10.3–15.7
	*Centropomus unionensis*			
*Macrostomorpha* spp.	*Bagre pinnimaculatus*	1	1.0 (SD±2.2)	0–4.2
*Methagonimus* spp.	*Centropomus unionensis*	8	7.7 (SD±1.3)	9.8–12.5
*Paragonimus* spp.	*Eugerres periche*	5	4.8 (SD±5.1)	0–9.8

*Eimeria* spp. and *Methagonimus* spp. were found in *Centropomus unionensis* at a prevalence of 12.5%, associated with *Biddulphia* spp. during the first sampling on March 19, 2023 (Table 2). However, this trend was not prominent during the subsequent sampling in October, where *Coscinodiscus* spp., *Navicula* spp., and *Paralia* spp. were present, and parasites were notably absent (Table 2, Figs 3, S2).

Interestingly, *Menticirrhus panamensis*, which tested negative for diatoms, was found parasitizing *Acanthocephalus* spp., whereas *Parapsettus panamensis*, *Bagre panamensis*, and *Lutjanus guttatus* tested negative for parasites, and exhibited associations with various diatoms, including *Coscinodiscus* spp., *Cymbella* spp., *Gyrosigma* spp., *Botrydiopsis* spp., *Melosira* spp., and *Torodinium* spp. (Table 2, Figs 4 and S2).

During the second sampling on October 07, 2023, samples from *Bagre pinnimaculatus* revealed infection with *Anisakis* eggs., along with the occurrence of ameboid cysts in association with *Aulacoseira* spp., *Botrydiopsis* spp., *Coscinodiscus* spp., and *Cyclotella* spp., at a prevalence of 20.7%. *Cynoscion albus* tested positive for parasites such as *Anisakis* spp. and *Balantidium* spp., and exhibited association with *Coscinodiscus* spp. Interestingly, *Mugil curema* showed the highest susceptibility to parasites across all samplings, with *Anisakis* spp., *Contracaecum* spp., and ameboid cysts (Table 2). Additionally, *Cynoscion albus* exhibited positivity for both *Anisakis* spp. and cilliated cyst., coinciding with the presence of *Coscinodiscus* spp. In contrast, *Cynoscion squamipinnis* and *Eugerres periche* were positive for *Coscinodiscus* spp. and *Navicula* spp., respectively. These showed negativity for parasites in the first and second samplings; however, *Eugerres periche* was present in diatoms such as *Aulacoseira* spp., *Coscinodiscus* spp, and *Paralia* spp. associated with *Paragonimus* spp, during the third sampling (Table 2, Figs 3 and 4 and S2).

*Notarius armbrusteri*, (Ñato sea-catfish) tested negative for parasites in October 2023, during the second samplings. However, it exhibited positivity for diatoms and parasites during the third sampling in March 2024, as it was Infected with *Acanthocephalus* spp., *Eimeria* spp., and ameboid cysts (Table 2, Figs 3 and 4 and S2).

*Thunnus alalunga* (longfin tuna), collected during the third sampling, exhibited a high prevalence of *Anisakis* spp. and *Contracaecum* spp. without any association with diatoms. Conversely, *Caranx sexfasciatus*, *Caranx vinctus*, *Caranx caninus*, *Lutjanus colorado*, *Nematistius pectoralis*, *Peprilus snyderi*, and *Strongylura fluviatilis* tested negative for diatoms and parasites across all samplings (Table 2, Figs 4, S2).

**Fig 5:**
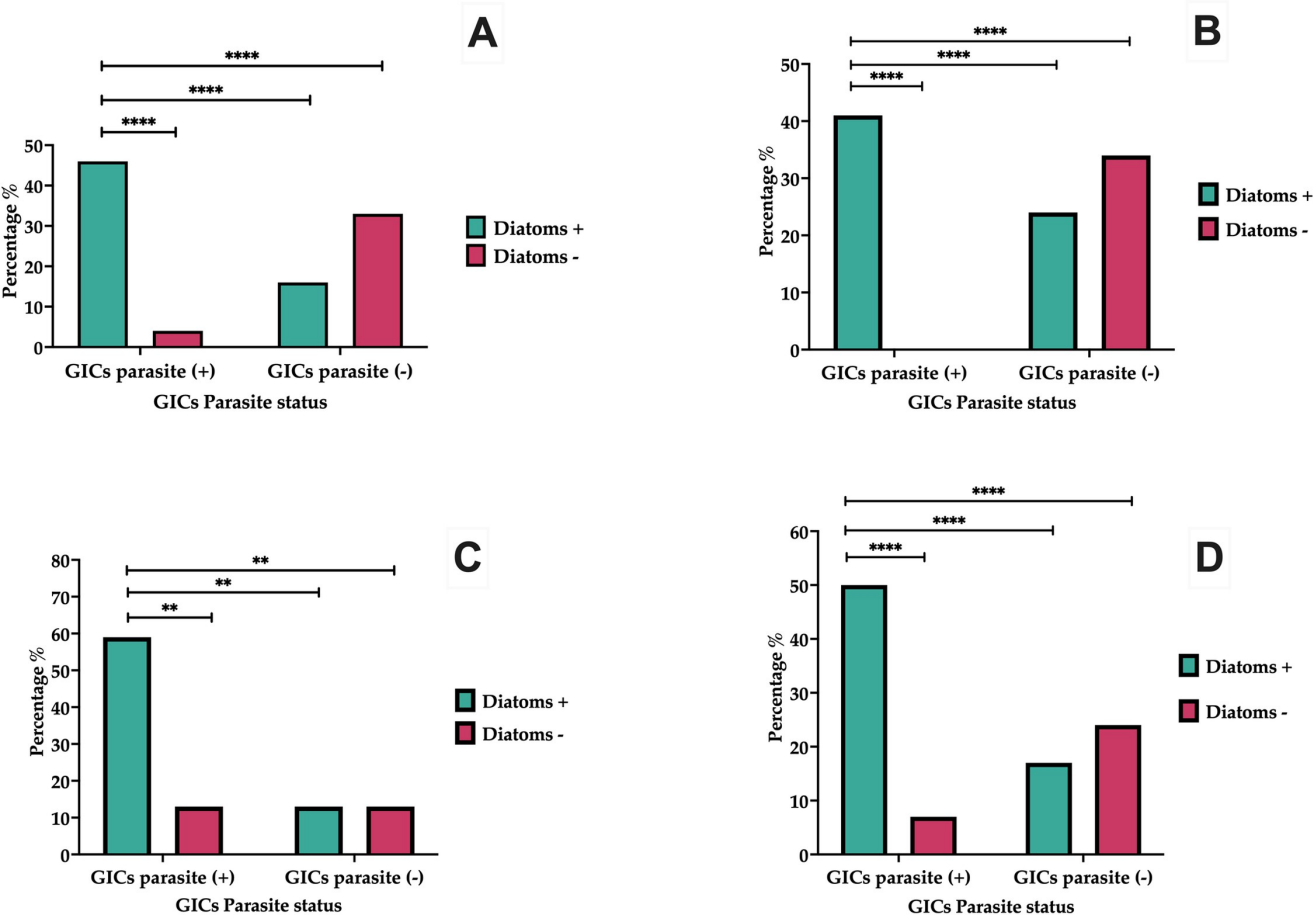
Contingency table. Parasites ‐ Diatoms association in Gastrointestinal Compartments (CGIs) in fish from harbor of Buenaventura–Colombia. First sampling 19 march 2023 (A), Second sampling 07 october 2023 (B), Third sampling 09 march 2024 (C), Total sampling (D). Significant differences using Fisher test P ≤0,0035, **. P≤0.0001****.

The association level between parasites and diatoms in each sampling was >1, with a 45.83% prevalence of parasites associated to diatoms (11 digestive compartments were positive for both), on March 19^th^, 2023. Only one (4.17%) gastrointestinal compartment was positive for parasites and negative for diatoms. Diatoms were present without parasites (16.67%). Conversely, digestive compartments without diatoms and parasites were 8 (33.3%). That means a factor association odds ratio with a value of 22.0, superior to >1. The presence of diatoms in the gastrointestinal compartments of fish is associated with a higher likelihood of parasitic infection. This is evident from the higher odds ratio (OR = 2.8) for compartments with diatoms, indicating that these compartments are significantly more likely to be associated to parasites compared to compartments without diatoms. Conversely, compartments without diatoms have a much lower likelihood of parasitic association (OR = 0.1), suggesting a protective effect or simply a lower association between the absence of diatoms and the presence of parasites. This analysis supports the conclusion that diatoms are correlated with an increased risk of parasitic infections in the gastrointestinal compartments of fish. (Fig 5, Table 3, S2 Fig).

**Table 3:** Measures of association, retrospective study cohort with percentage of grand total, relative risk and odds ratio.

**1st sampling (March 19.2023)**				
Dependent variable	CGI parasite (+)	CGI parasite (-)	Summatory	Odds ratio (OR)
Diatoms +	11 (45,83%)	4 (16,67)	15	2,8
Diatoms -	1 (4,17%)	8 (33,33%)	9	0,1
Total	12	12	24	22
**2nd sampling (October 07.2023)**				
Dependent variable	CGI parasite (+)	CGI parasite (-)	Summatory	Odds ratio (OR)
Diatoms +	12 (41,38%)	7 (24,14%)	19	1,7
Diatoms -	0 (0,00%)	10 (34,48%)	10	0
Total	12	17	29	1,7
**3rd sampling (March 09.2024)**				
Dependent variable	CGI parasite (+)	CGI parasite (-)	Summatory	Odds ratio (OR)
Diatoms +	30 (58,82%)	7 (13,73%)	37	4,3
Diatoms -	7 (13,73%)	7 (13,73%)	14	1
Total	37	14	51	4,3
**Full study**				
Dependent variable	CGI parasite (+)	CGI parasite (-)	Summatory	Odds ratio (OR)
Diatoms +	53 (50,96%)	18 (17,31%)	71	2.94
Diatoms -	8 (7,69%)	25 (24,04%)	33	0.32
Total	61	43	104	9.2

On October 7th, 2023, second sampling, the association between parasites and diatoms had a prevalence of 41.38%., in 12 compartments. No digestive compartments examined lacked both parasites and diatoms. Conversely, 24.14% (7) of the compartments harbored diatoms without parasites. The negative digestive compartments for diatoms and parasites were 34.48% (10). That means a factor association (Odds ratio) with a value of 1.7. The presence of diatoms in the gastrointestinal compartments of fish is associated with a higher likelihood of parasitic infection. However, the odds ratio of 1.7 suggests a moderate association compared to the first table, where the OR was 2.8. In contrast, compartments without diatoms showed no cases of parasitic infection (OR = 0.0), indicating that the absence of diatoms is strongly associated with the absence of parasitic infection. Overall, this data suggests that the presence of diatoms is associated to parasitic infection. (Table 3). In the last sampling, on March 9th, 2024, we found an association between parasites and diatoms in 30 digestive compartments from the total sampling (51), with a prevalence of 58.82%. 7 gastrointestinal compartments were positive for parasites and negative for diatoms (13.73%). There were 7 (13.73%) with diatoms present but without parasites. Finally, 7 digestive compartments were negative for diatoms and parasites (13.73%). Significant differences with high percentage of compartments with diatoms are positive for parasites compared to those without diatoms and a higher percentage of compartments without diatoms are negative for parasites. The presence of diatoms is strongly associated with a higher likelihood of parasitic infections using Fisher test P ≤0,0035, **. P≤0.0001****. (Fig 5, Table 3, S2 Fig).

## 4. Discussion

The presence of diatoms in the gastrointestinal compartments with strong associated with parasitic infection was demonstrated, as indicated by the high odds ratio of (OR:2,8; OR:1,7; OR:4,3; OR:2,94), this means that fishes with parasites are more likely to have compartments with diatoms compared to compartments without diatoms. In contrast, the compartments without diatoms have an equal likelihood of being infected or not infected by parasites (OR ≤ 1.0), suggesting no strong association between the absence of diatoms and parasitic infection. Overall, the data indicates a significant relationship between the presence of diatoms and parasitic infections in the fish’s gastrointestinal compartments and suggests that diatoms could be a contributing factor or an indicator of environments that is present when the parasitic infections are introduced.

The presence of diatoms in the gastrointestinal compartments of fish is associated with a higher likelihood of parasitic infection. An OR of 2.94 indicates that fish probably use diatoms when they become infected by parasites and parasites without parasitic infections has less probability to use diatoms, as indicated by the OR of 0.32. This suggests that the absence of diatoms is correlated with a reduced risk of parasitic infection (Fig 5). This data is associated to previous studies reporting the use of diatoms to control parasitic diseases in fish and reduce the use chemical treatments [5, 23]. Diatoms could play a role in facilitating or indicating environments where parasitic infections are more likely to occur and that the zoopharmacognosy might be a common phenomenoa in the ocean fish [25].

Nematodes of the Anisakidae family (genera Anisakis, Pseudoterranova and Contracaecum) are common fish parasites. Among these, *Anisakis simplex*, sensu stricto (s.s), and *Anisakis pegreffii* have been reported as causative agents infecting humans [51, 52]. This study identified various morphotypes of *Anisakis simplex* sensu lato, a larvae type I with an elongated ventricle and a mucron at the caudal end in Lisa (*Mugil curema*). This is consistent with previous reports of nematode larvae isolated in Cartagena (Colombia) from *Mugil incilis*, identified as *Contracaecum* spp., with a prevalence of 60.49% [37].

In Tumaco, Colombia, there have been reports of a type II *Pseudoterranova* larva, with a prevalence of 94%, identified as *A*. *physeteris* and *Pseudoterranova decipiens* [31]. *Contracaecum* sp. infection in *Hoplias malabaricus* (moncholo) were documented in rivers and marshes of Colombia, with a high intensity of 77.82% and a prevalence of 100%. Although the morphological and molecular diagnosis of parasites and diatoms was not incorporate [53].

*Anisakidae* eggs were found under coprology, ascarid nematodes divided into two distinct clades (families), the Anisakidae (which includes species of *Anisakis*, *Pseudoterranova* and *Contracaecum*) and the Raphidascarididae (which includes species of *Hysterothylacium* and *Raphidascaris*) [54]. The egg size of *A*. *simplex* was 41.3 μm ‐ 45.6 μm; *Pseudoterranova decipiens*: 45.2μm ‐ 41 μm; *Contracaecum osculatum*: 72.6 μm -80.9 μm [55]. Other studies had reported egg measures for *Contracaecum multipapillatum* s.l. of 53 × 43 μm, although after the larvae had developed inside, egg size increased to 66 × 55 μm [56], the egg of *Anisakis pegreffii* measure 40–60 μm in perimeter of egg oviposited [57] However, the size of the embryonated eggs can vary from one genus to another, so the morphological characteristics may present variations that make identification difficult [58]. This research found various Anisakidae eggs of varying sizes. These are compatible with *Contracaecum* spp. and *Anisakis* spp., considering the morphological description of adult L4 stages. Future molecular studies are required to identify the parasites at the level of specie.

Ciliated protozoa type *Balantidium* spp., primarily infects terrestrial animals, particularly pigs, and its presence in marine environments is not well-documented [34, 50]; However, there are other species within the *Balantidium* genus, such as *Balantidium polyvacuolum* and *Balantidium fulinensis*, which have been identified in fish [59, 60]. For example, *Balantidium polyvacuolum* has been observed in the hindgut of the fish species *Xenocypris davidi*, where it plays a role in energy metabolism by digesting plant material within the fish’s intestines [59, 60]. Here, we found a prevalence of ciliated type *Balantidium* spp. in fish gastrointestinal compartments of 6.7%, which is higher than previous reports in domestic and wild animals (0.89% to 4.17%) [34, 50]. Interestingly, 13 species of *Balantidium* spp., have been reported in fish, 4 of them in marine fish [61]. In South America, sea lions have been reported in *Otaria flavescens* with a prevalence of 13.8%, which represents an important biological component considering surveys on anthropozoonotic pathogens circulating in wild free-living sea lions and their possible impact on public health issues and marine wildlife [62]. The *B*. *ctenopharyngodoni* parasite was increasingly associated with bacterial diversity, a higher relative abundance of *Clostridium*, and a lower abundance of Enterobacteriaceae in grass carp, suggesting that the presence of *Balantidium ctenopharyngodoni* may improve intestinal health through changes in microbiota and metabolites [63]. *Balantidium piscicola* has been discovered parasitizing caranha *Piaractus brachypomus* and mandi catfish *Synodontis clarias* [64]. These findings suggest that while *Balantidium coli* may not commonly infect marine fish, other *Balantidium* species are adapted to aquatic environments and can be found in fish. This underscores the importance of accurately identifying the specific ciliate species involved when cysts are observed in marine fish using accurate molecular techniques.

Other parasites found in this study were three types of trematodes: *Methagonimus* spp. (with a prevalence of 7.7%), *Paragonimus* spp. (4.8%) and *Macrostomorpha* spp. (1%). Intestinal trematodes are taxonomically diverse and consist of more than 60 species worldwide. *Macrosotomorpha* spp., is a free-living flatworm, whose eggs are around 100 microns in diameter [65], with more than 100 marine species reported in fresh and brackish water. Species are contained in the genus *Macrostomum*, and some of them have worldwide distribution patterns [66]. They are an essential component of marine and freshwater ecosystems as top predators and secondary producers [67]. Among them, *Metagonimus* spp. is a source of human infection due to ingesting raw or improperly cooked fish. Its eggs are ovoid, pyriform, or elliptical with a size range of 21–35×12–21 μm [68, 69].

*Paragonimus* spp. constitutes a group of trematode parasites that infect humans throughout the world. Between 5 and 10% of Asia’s human population is infected. The areas with the highest possibility of infection are Asia, Central and South America, and Africa. An estimated 293 million people are at risk of infection [70]. These parasites have been reported in Colombia in Embera Indian communities located at the Colombian Pacific Coast and investigated in 1993–1998 [71]. Paragonimosis, is a lung disease characterized by symptoms such as coughing, haemoptysis, thoracic pain, and light dyspnea [71]. They are normally associated with the consume of undercooked freshwater crustaceans such as crab (*Moreirocarcinus emarginatus*) and primarily affect the lungs, but may ectopically migrate to other organs to produce a multisystemic clinical presentation [72]. In the present study *Paragonimus* spp. in fish are generally not considered common hosts; However, the polluted water in Buenaventura harbor could explain the unusual findings of these parasites, as contamination with fecal matter might contribute to their presence and Paragonimus as paratenic host [73–76].

In marine fishes, over 100 species of coccidians have been identified across 60 different fish families. These species are classified into five genera: Calyptospora and Crystallospora, each with one described species; Epieimeria, which includes four species; Eimeria, comprising 64 species; and Goussia, with 30 species [77].

The genus *Eimeria* consists of apicomplexan parasites primarily known for infecting terrestrial animals, especially birds and mammals, and causing coccidiosis. These parasites have a complex life cycle that includes both sexual and asexual reproduction phases, and they produce oocysts, which are excreted in the host’s feces. Eimeria is predominantly known for its impact on terrestrial animals, there are documented cases of various Eimeria species in fish, both freshwater and marine. Numerous species of Eimeria have indeed been identified in fish, including marine species. Some examples include: *Eimeria adioryxi* (reported in marine fish), *Eimeria anguillae* (found in eels), *Eimeria dingleyi* (found in marine fish), *Eimeria dicentrarchi* (documented in marine environments), *Eimeria catalina* and others. This diversity suggests that while Eimeria is more commonly associated with terrestrial hosts, it also has representatives in aquatic environments, including marine fish [77].

In the present study, coccidias type *Eimerias* spp. was identified in *Notarius armbrusteri* and *Centropomus unionensis*, at a prevalence of 10.6%. Multiple species of coccidians (Apicomplexa: Eimeriorina) from the *Epieimeria*, *Calyptospora*, and *Crystallospora* genera have been reported in marine teleosts; some of them have contribuited to high mortality [77, 78]. The presence of coccidia species in *Centropomus unionensis*, *Notarious armbrusteri*, in Colombia represents an important field of study that needs to be understood. In some of these species, parasites’ intestinal epithelial cells are temperature dependent, where their development is complete at 20°C in 9 to 10 d post-exposure (PE), but the metabolism is reduced at low temperatures, at 8 to 10°C 37 d (PE). Probably, the presence of these parasites is related to temperature averages (March 2023, 26,6^o^; March 2024, 28°C). Future studies incorporating molecular techniques will help to elucidate accurately the genus and species of Eimerias in this ecosystems.

*Acanthocephalus* spp. had a prevalence of 8.7%, associated to *Notarius armbrusteri* (Spanish common name: ñato), and *Menticirrhus panamensis* (Spanish common name: botellona). *Acanthocephalus* spp. are endoparasites of the vertebrate intestine and have a heteroxenous life cycle with at least one arthropod intermediate host In their larval stages. They comprise 1200 species that measure a few millimeters in length; others are larger in size, such as *Macracanthorhynchus hirudinaceus* (7–15 cm). Some reported species in fish are *Echinorhynchus truttae* (0,7–2,2 mm) *Salmo* sp., with *Gammarus* sp. (Amphipoda) as intermediate host; *Pomphorhynchus laevis* (1–3.5 mm) (*Gammarus* sp.—Amphipoda—intermediate host); and *Neoechinorhynchus rutili* 0.5–1 in *Cyprinus carpio* as definitive host, and Ostracoda as intermediate host [79–82]. More than 50 Acanthocephalan species have been reported in the Pacific Oceans from freshwater and marine fish, amphibians, reptiles, birds, and mammals such as *Rhadinorhynchus circumspinus* n. sp., *Rhadinorhynchus pacificus* n. sp., *Rhadinorhynchus multispinosus* n. sp. [83].

In Mexico, *Acanthocephalus amini* n. sp. (Palaeacanthocephala: Echinorhynchidae) has been documented in the intestine of *Cichlasoma urophthalmus* (Günther) (Pisces: Cichlidae), collected in Río Champotón, a river in Campeche State, Mexico [84]. In Peru, there have been 71 described species in fish [85], whereas the literature documents 23 genera in Brazil, comprising 34 named species and 13 undetermined species of acanthocephalans, parasitizing Brazilian fishes [86]. Unfortunately, Costa Rica, Venezuela, Colombia, Chile, and Uruguay exhibited the lowest publication numbers, resulting in gaps in the distribution of acanthocephalans [87].

Regarding diatoms, we found 14 genus of diatoms in the intestinal compartments of 22 fish species. This is a normal value, but it is also lower than those expressed In previous reports in the Coast zones of Caribbean oceans, which have reported 337 taxon, 312 species [88]. In the Pacific Ocean coast of Colombia, San Andres de Tumaco, has been reported 101 genus and 262 species of diatoms [89]. This is the first report of diatoms in gastrointestinal compartments in various species of fish on the Pacific coast of Colombia. There are several studies covering phytoplankton species, especially regarding diatoms in the Buenaventura harbor. Usually, Buenaventura reports less species than Tumaco, which can be likely attributed to salinity, port harbor activity, and contamination levels [90]. These findings can explain why the species of diatoms in the present study was lower than in previous reports.

Diatoms are unicellular algae made of siliceous skeletons called frustules and are found in almost every aquatic environment, including fresh and marine waters [91]. Here, we found a strong association of parasites and diatoms, a value of 9.2, which is >1. (Table 3). Of 104 digestive compartments, 53 tested positives for diatoms and parasites, indicating a prevalence rate of 51%. Parasites were detected in 8 compartments, but in these Instances diatoms were absent, accounting for 7.7% of the cases. Conversely, 18 compartments tested positive for diatoms but negative for parasites, representing 17.3% of the total. Additionally, 25 compartments showed negative results for both diatoms and parasites, making up 24% of the samples.

Among the identified diatoms, *Coscinodiscus* spp. exhibited the highest prevalence, at 55.8%, indicating its dominance in the dietary diatom frequency within the gut content. These findings suggest that the genus *Coscinodiscus* significantly contributes to the composition of diatoms in the gut. Moreover, previous studies support our results by identifying four distinct species of *Coscinodiscus* and confirming their taxonomic classification based on SEM-based characters [7].

Some diatoms groups usually form colonies [92]. Free-living diatoms are covered by a siliceous skeleton (frustule) composed of SiO2 and H2O [92]. The structure of the frustule is the main feature used to identify species [93].

Marine diatoms have diverse bioactive molecules and have a great value for the nutraceutical industry, with compounds such as carotenoids, proteins, vitamins, essential amino acids, and omega-rich oils [8, 94]. Marine diatoms have shown promising potential as agents with anti-parasitic effects. These effects stem from various bioactive compounds produced by diatoms, which exhibit cytotoxic, anti-inflammatory, and immunomodulatory properties. Silica shells produce a mechanical disruption, provide structural support and possess abrasive properties. The rigid silica shells of diatoms can mechanically disrupt parasites, including protozoa and helminths, upon contact. This mechanical disruption can interfere with the attachment, feeding, and reproduction of parasites, thereby limiting their ability to establish infections [95].

Diatoms also produce a diverse array of bioactive compounds, including polyunsaturated fatty acids (PUFAs), sterols, alkaloids, and phenolic compounds, which exhibit anti-parasitic activity. For example, PUFAs such as eicosapentaenoic acid (EPA) and docosahexaenoic acid (DHA) have been shown to disrupt the lipid metabolism of parasites, leading to membrane damage and impaired viability. Similarly, sterols present in diatoms can interfere with parasite cell membrane integrity, disrupting essential physiological processes [96].

Some diatom-derived compounds also possess immunomodulatory properties, which can enhance the host’s immune response against parasitic infections. For instance, polysaccharides isolated from certain diatom species have been shown to stimulate macrophage activity and cytokine production, thereby promoting the clearance of parasites by the host immune system. Furthermore, diatom-derived antioxidants can mitigate inflammation associated with parasitic infections, reducing tissue damage and facilitating the resolution of infection [97]. Additionally, some diatom species produce antimicrobial peptides (AMPs) as part of their defense mechanisms against microbial pathogens, which may also exhibit activity against parasitic organisms. These peptides can disrupt parasite cell membranes, interfere with essential metabolic pathways, or modulate host immune responses to combat parasitic infections. Research in this area is ongoing, with efforts focused on identifying and characterizing novel diatom-derived AMPs with potent anti-parasitic properties [98]. Diatomaceous earth has been found useful in the prevention of worm infestation in purebred pigeons [99]. Recent studies have supported its use as animal growth promoter, vaccine adjuvant in livestock, water purifier, mycotoxin binder, inert dust applications in stored-pest management, pesticide, animal feed additive, as a natural source of silicon in livestock, and as natural anthelmintic [100]. Fossil shell flour has the potential to supplement traditional crops in beef cattle rations in response to complex global challenges, since it Is cheap, readily available, and eco-friendly. However, it has not gained much attention from scientists, researchers, and farmers, and its use has not yet been adopted in most countries [101]. For the past two decades, fossil shell flour (FSF) has been used to naturally deworm animals, as 2% inclusion rate of FSF can be used with positive results in the destruction of internal parasites and worms [100].

In the present study, we did not capture fish for our research methodology. To collect the gastrointestinal tracts, we followed the cultural fish practice developed by artisanal fisheries of the municipality of Buenaventura (Pacific of Colombia). This cultural practice is characterized by species that inhabit coastal habitats such as mangroves and a limited number of pelagic species (tuna, sawfish, billfish, pipefish). These fisheries represent >21% of all fishery landings in the region. Normally, communities capture around 12 species exclusively by trammel net. Some of the captured species are herbivorous and therefore can only be caught with this process. Trammel nets also have the ability to capture a more diverse collection of species, although not necessarily those of commercial value [46]. The most common species caught using trammel nets in the artisanal fisheries of Buenaventura are *Bagre pannamensis*, *Caulolatilus affinis*, *Cyncoscion albus*, *Notarius troscheli*, *Centropomus viridis*, *Bagre pinnimaculatus*, *Centropomus medius*, *Cynoscion reticulatus*, *Oligoplites altus*, *Centropomus* sp. Some areas have reported up to 38 species of fish richness [46]. In the present research, we found 22 species of fish in the area, This implies that means a diverse array of present species within the community or ecosystem. This also indicates a healthy and varied environment, supporting a wide range of organisms.

The individuals within the community are distributed relatively evenly among the different species. This means that not only there are a significant amount of present species, but they are also well-balanced in terms of population sizes. Higher number of species often correlates with ecosystem stability. That mean that the ecosystem is resilient to disturbances due to the fact that it has a variety of species that can fulfill different roles and functions. This resilience can help the ecosystem withstand environmental changes and maintain its structure and function over time, which is often considered desirable for conservation and ecological sustainability.

Species of fish such as *Cynoscion albus* were *positive for Anisakis* spp. *Balantidium* spp. and diatoms such as *Coscinodiscus* spp. This species is distributed from Mexico to Ecuador and it is an important meat predator of second order; however, there is minimal information about its food habits [102]. This species live between 0–41 m. of depth [103]. The yellow croaker *Cynoscion albus* is a species that is distributed in the eastern Pacific from Baja California to Peru. It inhabits coastal waters, while juveniles enter shallow bays, estuaries, and river mouths. It is a bento-pelagic species. It feeds on shrimp, crabs, cephalopods and fish. It reaches sizes of up to 130 cm total length (TL) [104].

The lisa fish (*Mugil Curema*) is distributed in the Pacific ocean and inhabis water coasts at a depth rangin from 0 to 15 m. It is an omnivorous fish that consumes insects and algae; for this reason, they are an intermediate parasite host, contributing to the development of anisakis stages. In this species has been found the presence of abundant diatoms such as *Gyrosigma*, *Navicula*, *Cymbella*, *Fragilaria*, and *Nitzschia* [105]. This study found other additional diatoms such as *Coscinodiscus*, *Botrydiopsis*, *Cyclotella*, *Melosira*, *Paralia*, *Skeletonema*, and *Unruhdinium*. We confirmed the presence of *Gyrosigma* and *Navicula*. Other studies developed in Mexico reported 130 taxa dominating *Nitzschia*, *Navicula*, *Amphora*, and *Cocconeis* dominated [106]. To our knowledge, there are no previous reports of diatoms for these species associated to parasites. Therefore, we present the first study reporting the association of diatoms and parasites in gastrointestinal GI tracts.

Although species such as Tuna *Thunnus alalunga* did not report the presence of diatoms, fish that are primarily carnivorous or piscivorous (fish-eating) may not actively seek out diatoms as a significant portion of their diet. Instead, they may prefer other prey such as small fish, crustaceans, or insects. Interestingly, we found a significant parasitic charge of parasites that may be associated to the carnivorous and migratory habits as pelagic fish which is distributed in most tropical and temperate oceans [107, 108]. In Colombia, *Thunnus alalunga* has a wide distribution in the Colombian Caribbean (depth range: 0 to 600 m.). The diet of adults comprises a variety of fish, squid and crustaceans, while the diet of juveniles comprises mainly fish (anchovies, saury, sardines, juvenile hake) [109]. In the case of parasites, at least 14 valid species parasitizing *T*. *alalunga* have been reported as metazoan gill parasite species, isopods, copepods, nematodes, trematodes, and acanthocephalans (*Echinorhynchus* sp.) [110–112]. In Brazil, the presence of nematodes of the Anisakidae family is recorded: *A*. *simplex* in *T*. *albacares*, *T*. *atlanticus* and *T*. *obesus*, of *A*. *physeteris* in *T*. *albacares*, of *Contracaecum* sp. in *T*. *atlanticus* and *T*. *obesus* and *Raphidascaris* sp. in *T*. *albacares* and *T*. *obesus*. [113].

*Lutjanus guttatus* is a fish that we found harboring eggs of *Metagonimus* spp., in its gastrointestinal tracts, but was negative for diatoms, which is consistent with its carnivorous diet. It acts as final or intermediate host for several parasite species, throughout its life cycle [114].

*Lutjanus guttatus* ranges from the Gulf of California to northern Peru, encompassing the Galapagos Islands. Despite being subject to high levels of exploitation, it is categorized by the International Union for Conservation of Nature (IUCN) as being in the least concern category [115]. It is a benthopelagic species, present in marine and brackish habitats, associated with reefs and shallow waters. It is a carnivorous species, feeding on invertebrates and fish. As an adult it lives in coastal reefs, up to about 30 m. deep, and during its youth in estuaries and river mouths. It is a carnivorous species that feeds on fish and invertebrates [116]. It is an important resource for artisanal fishermen on the Mexican Pacific coast. In this fish there have been identified 32 taxa of metazoan parasites: four species of Digenea, four Monogenea, one Cestoda, two Acanthocephala, seven Nematoda (the nematodes Anisakis sp., Hysterothylacium sp. and Procamallanus sp.), one Hirudinea and nine Crustacea (six Copepoda and three Isopoda).

The outcomes of our study were impacted by climatic conditions, including global warming and phenomena like El Niño and La Niña. We concur with Villalba’s perspective that the dynamics of regional and local marine parasites could be influenced by these factors [114]. Changes in dietary preferences, such as increased consumption of raw fish and undercooked fish products, alongside evolving food habits and tastes, may elevate the likelihood of consumers being exposed to parasitic risks [61]. The risk can be mitigated by storing the fish at temperatures of −20°C or below for a total of seven days, or at −35°C or below for 15 hours. Additionally, ensuring proper preparation of dishes by thoroughly cooking the food at a temperature of 60°C for at least 10 minutes can help reduce the risk. However, these practices are not consistently followed in various communities [17, 117, 118].

The widespread occurrence and significant correlation of certain diatoms, including species like *Cymbella* spp., *Aulacoseira* spp., *Coscinodiscus* spp., *Gyrosigma* spp., *Navicula* spp., and *Paralia* spp., alongside the presence of parasites in various fish species, lead us to hypothesize that fish might acquire these diatoms as a form of nutraceutical treatment. This speculation arises from observations such as the application of *P*. *tricornutum* as an antiparasitic remedy for monogenean diseases in aquaculture [5, 8]. *Coscinodiscus spp*., along with other diatoms mentioned in this research, are promising subjects for further investigation, as previous studies have noted that *Coscinodiscus* is a prevalent component of the diet in the gut content studies [7].

Finally, this work consider that this research delved into the intricate relationship between diatoms and parasites in the gastrointestinal tracts of various fish species inhabiting the Buenaventura harbor along the Pacific Ocean. Through coprological techniques and microscopic examinations, we were able to identify a diverse array of diatoms and parasites, shedding light on their associations and prevalence rates. Notably, we observed zoonotic parasites like *Anisakis* spp., *Acanthocephalus* spp., and *Contracaecum* spp., alongside protozoa like *Balantidium* spp. Furthermore, we found several species of trematodes and coccidians, enriching our understanding of the parasite diversity within these fish populations. Our study also revealed insights into the dietary habits and ecological roles of these fish species, with some showing preferences for specific prey items and habitats. Additionally, the presence of certain diatom species in the gut content suggests a potential role in fish nutrition and health, which warrants further investigation.

Climate change and phenomena such as El Niño and La Niña were identified as factors influencing the dynamics of marine parasites, highlighting the need for ongoing monitoring and adaptation strategies [119]. Moreover, the risks associated with consuming raw or undercooked fish underscore the importance of proper food handling and preparation practices to mitigate parasitic infections. Overall, our findings hope to contribute to the broader understanding of marine species richness and ecosystem dynamics, emphasizing the interconnectedness of species within these complex environments. Future research incorporating morphological and molecular techniques could provide deeper insights into the interactions between diatoms, parasites, and their hosts, paving the way for enhanced strategies in parasite management and ecosystem conservation.

### 4.1 Limitations of the study

The study has two important limitations to consider when interpreting the results. Firstly, the Port of Buenaventura is restricted during March–April, which prohibits the capture and commercialization of fish, following Colombia’s regulations that aim to facilitate fish reproduction and preserve its species richness. The sample collection was randomly collected and, depending on the season and year, it may not be possible to collect exactly the same number of gastrointestinal compartment in each period of time. Secondly, some areas were difficult to access and are located in distant territories. And finally in spite of having sampled all units (fish), the sample size is too small to find some relationship or differences. Nevertheless, it is not reasonable to discard its hypothetical effects.

## 5. Conclusions

The GICs analysis of fish provided useful insights regarding its diatoms associated with parasites as a first report in Colombia. The genus *Coscinodiscus* spp. and *Gyrosigma* spp. dominated the diatom frequency during the study in different compartments associated with a diversity of fish using optical microscopy and SEM. Diatoms not only have nutritional profiles for fish but also, in some trophic levels, they are strongly associated with parasites, especially *Mugil curema*. This study also opens an avenue for research in searching for diatoms as preferential fish feed and their further screening for the purpose of identifying a commercially viable bioactive compounds for formulating potential value-added nutraceuticals and supplementary food for various dietary requirements of humans, livestock, and in aquaculture. Finally, future studies need to incorporate morphological and molecular diagnosis of parasites and diatoms.

## Supporting information

S1 Fig First, second and third sampling. Notes the different species of diatoms found in the fish species collected from Buenaventura harbor -Colombia.
(DOCX)

S2 Fig Different time sampling. Association of parasites and diatoms positive in fish sampling.
(DOCX)

S1 Table Diatoms and parasites are found in the gastrointestinal compartments of the fish population in Buenaventura port of Colombia. (DOCX)

## References

[1] Mann DG, Vanormelingen P. An inordinate fondness? The number, distributions, and origins of diatom species. Journal of Eukaryotic Microbiology. 2013;60(4):414–20. Epub 20130527. doi: 10.1111/jeu.12047 . 23710621

[2] Dahiya P, Makwana MD, Chaniyara P, Bhatia A. A comprehensive review of forensic diatomology: contemporary developments and future trajectories. Egyptian Journal of Forensic Sciences. 2024;14(1). doi: 10.1186/s41935-023-00378-7

[3] Benito X, Fritz SC. Diatom Diversity and Biogeography Across Tropical South America. Neotropical Diversification: Patterns and Processes Fascinating Life Sciences Springer. 2020. doi: 10.1007/978-3-030-31167-4_7

[4] Rivera MJ, Luis AT, Grande JA, Sarmiento AM, Davila JM, Fortes JC, et al. Physico-Chemical Influence of Surface Water Contaminated by Acid Mine Drainage on the Populations of Diatoms in Dams (Iberian Pyrite Belt, SW Spain). International Journal of Environmental Research and Public Health. 2019;16(22). Epub 20191115. doi: 10.3390/ijerph16224516 ; PubMed Central PMCID: PMC6888037. 31731686 PMC6888037

[5] Kim J-H, Didi-Cohen S, Khozin-Goldberg I, Zilberg D. Translating the diatom-grazer defense mechanism to antiparasitic treatment for monogenean infection in guppies. Algal Research. 2021;58. doi: 10.1016/j.algal.2021.102426

[6] Saxena A, Kumar Singh P, Bhatnagar A, Tiwari A. Growth of marine diatoms on aquaculture wastewater supplemented with nanosilica. Bioresour Technol. 2022;344(Pt A):126210. Epub 20211026. doi: 10.1016/j.biortech.2021.126210 . 34715335

[7] Choudhury S, Basuli D, Das T, Nandi S, Sarkar NS. Exploring fatty acid connections between estuarine fish Chelon planiceps and its diatom diet as taste and nutraceutical property influencing factor. Algal Research. 2023;72. doi: 10.1016/j.algal.2023.103116

[8] Nieri P, Carpi S, Esposito R, Costantini M, Zupo V. Bioactive Molecules from Marine Diatoms and Their Value for the Nutraceutical Industry. Nutrients. 2023;15(2). Epub 20230116. doi: 10.3390/nu15020464 ; PubMed Central PMCID: PMC9861441. 36678334 PMC9861441

[9] Round FE, Crawford RM, Mann DG. The diatoms: biology and morphology of the genera, ix, 747p. Cambridge University Press, 1990. Price £125.00. Journal of the Marine Biological Association of the United Kingdom. 2009;70(4):924-. doi: 10.1017/s0025315400059245

[10] Cordero del Campillo M. Parasitología veterinaria: McGraw-Hill Interamericana; 2000.

[11] Bellay S, EF DEO, Almeida-Neto M, Mello MA, Takemoto RM, Luque JL. Ectoparasites and endoparasites of fish form networks with different structures. Parasitology. 2015;142(7):901–9. Epub 20150316. doi: 10.1017/S0031182015000128 . 25774533

[12] Shinn AP, Pratoomyot J, Bron JE, Paladini G, Brooker EE, Brooker AJ. Economic costs of protistan and metazoan parasites to global mariculture. Parasitology. 2015;142(1):196–270. Epub 20141202. doi: 10.1017/S0031182014001437 . 25438750

[13] da Rocha CAM. Parasitic Helminths of the Freshwater Neotropical Fish Hoplias malabaricus (Characiformes, Erythrinidae) from South America Basins. Reviews in Fisheries Science. 2011;19(2):150–6. doi: 10.1080/10641262.2011.557752

[14] Costello C, Ovando D, Clavelle T, Strauss CK, Hilborn R, Melnychuk MC, et al. Global fishery prospects under contrasting management regimes. Proc Natl Acad Sci U S A. 2016;113(18):5125–9. Epub 20160328. doi: 10.1073/pnas.1520420113 ; PubMed Central PMCID: PMC4983844. 27035953 PMC4983844

[15] Darwall WR, Freyhof J. Lost fishes, who is counting? The extent of the threat to freshwater fish biodiversity. Conservation of freshwater fishes. 2016:1–36.

[16] Bellay S, de Oliveira EF, Almeida-Neto M, Takemoto RM. Ectoparasites are more vulnerable to host extinction than co-occurring endoparasites: evidence from metazoan parasites of freshwater and marine fishes. Hydrobiologia. 2020;847(13):2873–82. doi: 10.1007/s10750-020-04279-x

[17] Eiras JC, Pavanelli GC, Takemoto RM, Nawa Y. Fish-borne nematodiases in South America: neglected emerging diseases. J Helminthol. 2018;92(6):649–54. Epub 20171025. doi: 10.1017/S0022149X17001006 29067898

[18] Jurado LF, Palacios DM, Lopez R, Baldion M, Matijasevic E. [Cutaneous gnathostomiasis, first confirmed case in Colombia]. Biomedica. 2015;35(4):462–70. doi: 10.7705/biomedica.v35i4.2547 26844434

[19] Theunissen C, Bottieau E, Van Gompel A, Siozopoulou V, Bradbury RS. Presumptive Gnathostoma binucleatum-infection in a Belgian traveler returning from South America. Travel Med Infect Dis. 2016;14(2):170–1. Epub 20160304. doi: 10.1016/j.tmaid.2016.02.003 . 26960751

[20] Bennett DC, Yee A, Rhee YJ, Cheng KM. Effect of diatomaceous earth on parasite load, egg production, and egg quality of free-range organic laying hens. Poult Sci. 2011;90(7):1416–26. doi: 10.3382/ps.2010-01256 . 21673156

[21] Simon CA, Bentley MG, Caldwell GS. 2,4-Decadienal: Exploring a novel approach for the control of polychaete pests on cultured abalone. Aquaculture. 2010;310(1–2):52–60. doi: 10.1016/j.aquaculture.2010.10.031

[22] Troncoso J, Gonzalez J, Pino J, Ruohonen K, El Mowafi A, Gonzalez J, et al. Effect of polyunsatured aldehyde (A3) as an antiparasitary ingredient of Caligus rogercresseyi in the feed of Atlantic salmon, Salmo salar. Latin American Journal of Aquatic Research. 2011;39(3):439–48. doi: 10.3856/vol39-issue3-fulltext-5

[23] Jawaji A, Goldberg IK, Zilberg D. Exploring the use of fatty acid ethyl esters as a potential natural solution for the treatment of fish parasitic diseases. J Fish Dis. 2024:e13991. Epub 20240629. doi: 10.1111/jfd.13991 . 38943443

[24] Díaz JM, Acero AP. Marine biodiversity in Colombia: Achievements, status of knowledge, and challenges. Gayana. 2003;67:261–74. doi: 10.4067/S0717-65382003000200011

[25] Vaughan DB, Saunders RJ, Hutson KS. How do fishes manage disease? Trends Ecol Evol. 2023;38(5):396–8. Epub 20230211. doi: 10.1016/j.tree.2023.01.017 . 36775796

[26] Dominguez-Martin EM, Tavares J, Rijo P, Diaz-Lanza AM. Zoopharmacology: A Way to Discover New Cancer Treatments. Biomolecules. 2020;10(6). Epub 20200526. doi: 10.3390/biom10060817 ; PubMed Central PMCID: PMC7356688. 32466543 PMC7356688

[27] Selvaraj JJ, Guerrero D, Cifuentes-Ossa MA, Guzman Alvis AI. The economic vulnerability of fishing households to climate change in the south Pacific region of Colombia. Heliyon. 2022;8(5):e09425. Epub 20220513. doi: 10.1016/j.heliyon.2022.e09425 ; PubMed Central PMCID: PMC9126920. 35620620 PMC9126920

[28] Moreno LT. La Pesca E Los Pescadores Artesanales En Colombia. PEGADA ‐ A Revista da Geografia do Trabalho. 2018;19(2). doi: 10.33026/peg.v19i2.5514

[29] Pochet Ballester GI. Ley Nº 2268 del 3 de agosto de 2022 de Colombia: un análisis desde las Directrices voluntarias sobre pesca artesanal de la FAO. Revista de la Facultad de Derecho de México. 2023;73(286):679–700. doi: 10.22201/fder.24488933e.2023.286.86404

[30] Gil-Agudelo D, Espinosa S, Delgado M, Gualteros W, Lucero C. La pesquería tradicional de piangua en el Pacífico colombiano, entre la subsistencia y el comercio. 2011. p. 49–79.

[31] Jenniffer Alejandra Castellanos G, Rubén Mercado P, Sebastián Peña F, María Carolina Pustovrh R, Liliana Salazar M. Anisakis physeteris y Pseudoterranova decipiens en el pez Mugil curema comercializado en Tumaco, Colombia. Revista MVZ Córdoba. 2020;25(2). doi: 10.21897/rmvz.1781

[32] Velez N, Bessudo S, Barragan-Barrera DC, Ladino F, Bustamante P, Luna-Acosta A. Mercury concentrations and trophic relations in sharks of the Pacific Ocean of Colombia. Mar Pollut Bull. 2021;173(Pt B):113109. Epub 20211105. doi: 10.1016/j.marpolbul.2021.113109 . 34749115

[33] Duque G, Gamboa-Garcia DE, Molina A, Cogua P. Effect of water quality variation on fish assemblages in an anthropogenically impacted tropical estuary, Colombian Pacific. Environ Sci Pollut Res Int. 2020;27(20):25740–53. Epub 20200430. doi: 10.1007/s11356-020-08971-2 ; PubMed Central PMCID: PMC7329768. 32356057 PMC7329768

[34] Zárate Rodriguez PT, Collazos-Escobar LF, Benavides-Montaño JA. Endoparasites Infecting Domestic Animals and Spectacled Bears (Tremarctos ornatus) in the Rural High Mountains of Colombia. Veterinary Sciences. 2022;9(10). doi: 10.3390/vetsci9100537 36288150 PMC9608847

[35] Bush AO, Lafferty KD, Lotz JM, Shostak AW. Parasitology meets ecologyon its own terms: Margolis et al revisited. The Journal of Parasitology. 1997;83(4). 9267395

[36] Margolis L, Esch G.W., Holmes J.C., Kuris M., Schad G.A., The use of ecological terms in parasitology (report of an ad hoc committee of the American Society of Parasitologists. J Parasitol. 1982;68:131–3.

[37] Olivero-Verbel J, Baldiris-Avila R, Arroyo-Salgado B. Nematode infection in Mugil incilis (Lisa) from Cartagena Bay and Totumo Marsh, north of Colombia. J Parasitol. 2005;91(5):1109–12. doi: 10.1645/GE-392R1.1 . 16419755

[38] Bush AO, Lafferty KD, Lotz JM, Shostak AW. Parasitology meets ecology on its own terms: Margolis et al. revisited. J Parasitol. 1997;83(4):575–83. . 9267395

[39] Anderson RC. Nematode Parasites of Vertebrates their Development and Transmission. CABI Publishing, Wallingford, Oxon, UK. 2000:650.

[40] Verbel JO, Caballero-Gallardo K, Arroyo-Salgado B. Nematode infection in fish from Cartagena Bay, North of Colombia. Vet Parasitol. 2011;177(1–2):119–26. Epub 20101119. doi: 10.1016/j.vetpar.2010.11.016 . 21168279

[41] Reichenbach-Klinke HH. Claves para el diagnóstico de las enfermedades de los peces Zaragoza ‐ España: Acribia; 1975.

[42] Melamed A, Robinson JN. A study design to identify associations: Study design: observational cohort studies. BJOG. 2018;125(13):1776. Epub 20180619. doi: 10.1111/1471-0528.15203 . 29916202

[43] Klebanoff MA, Snowden JM. Historical (retrospective) cohort studies and other epidemiologic study designs in perinatal research. Am J Obstet Gynecol. 2018;219(5):447–50. Epub 20180905. doi: 10.1016/j.ajog.2018.08.044 . 30194051

[44] Meneguetti DUDO, Silva RPM, Camargo LMA. Research methodology topics: Cohort studies or prospective and retrospective cohort studies. Journal of Human Growth and Development. 2019;29(3):433–6. doi: 10.7322/jhgd.v29.9543

[45] Olivero-Verbel J, Baldiris-Avila R, Guette-Fernandez J, Benavides-Alvarez A, Mercado-Camargo J, Arroyo-Salgado B. Contracaecum sp. infection in Hoplias malabaricus (moncholo) from rivers and marshes of Colombia. Vet Parasitol. 2006;140(1–2):90–7. Epub 20060502. doi: 10.1016/j.vetpar.2006.03.014 . 16650597

[46] Tilley A, Box S. Análisis de Vulnerabilidad de las Pesquerías Artesanales del Municipio de Buenaventura, Pacífico Colombiano (USAID, BIOREDD+)2014.

[47] Chasqui Velasco AP, Arturo Acero, Paola A. Mejía-Falla, Andrés Navia, and Luis Alonso Zapata. Libro rojo de peces marions de Colombia2017.

[48] Cribb TH, Bray RA. Gut wash, body soak, blender and heat-fixation: approaches to the effective collection, fixation and preservation of trematodes of fishes. Syst Parasitol. 2010;76(1):1–7. Epub 20100417. doi: 10.1007/s11230-010-9229-z . 20401574

[49] Narvaez P, Yong RQ-Y, Grutter AS, Hutson KS. Are cleaner fish clean? Marine Biology. 2021;168(5). doi: 10.1007/s00227-021-03858-3

[50] Pena-Quistial MG, Benavides-Montano JA, Duque NJR, Benavides-Montano GA. Prevalence and associated risk factors of Intestinal parasites in rural high-mountain communities of the Valle del Cauca-Colombia. PLoS Negl Trop Dis. 2020;14(10):e0008734. Epub 20201009. doi: 10.1371/journal.pntd.0008734 ; PubMed Central PMCID: PMC7591239. 33035233 PMC7591239

[51] Martin-Carrillo N, Garcia-Livia K, Baz-Gonzalez E, Abreu-Acosta N, Dorta-Guerra R, Valladares B, et al. Morphological and Molecular Identification of Anisakis spp. (Nematoda: Anisakidae) in Commercial Fish from the Canary Islands Coast (Spain): Epidemiological Data. Animals (Basel). 2022;12(19). Epub 20220930. doi: 10.3390/ani12192634 ; PubMed Central PMCID: PMC9559264. 36230375 PMC9559264

[52] Chang T, Jung BK, Hong S, Shin H, Lee J, Patarwut L, et al. Anisakid Larvae from Anchovies in the South Coast of Korea. Korean J Parasitol. 2019;57(6):699–704. Epub 20191231. doi: 10.3347/kjp.2019.57.6.699 ; PubMed Central PMCID: PMC6960240. 31914524 PMC6960240

[53] Borges JN, Cunha LF, Santos HL, Monteiro-Neto C, Portes Santos C. Morphological and molecular diagnosis of anisakid nematode larvae from cutlassfish (Trichiurus lepturus) off the coast of Rio de Janeiro, Brazil. PLoS One. 2012;7(7):e40447. Epub 20120709. doi: 10.1371/journal.pone.0040447 ; PubMed Central PMCID: PMC3392247. 22792329 PMC3392247

[54] Chen HX, Zhang LP, Gibson DI, Lu L, Xu Z, Li HT, et al. Detection of ascaridoid nematode parasites in the important marine food-fish Conger myriaster (Brevoort) (Anguilliformes: Congridae) from the Zhoushan Fishery, China. Parasit Vectors. 2018;11(1):274. Epub 20180502. doi: 10.1186/s13071-018-2850-4 ; PubMed Central PMCID: PMC5930788. 29716661 PMC5930788

[55] Victoria Herreras M E. Montero F, J. Marcogliese D, Antonio Raga J, A. Balbuena J. Phenotypic tradeoffs between egg number and egg size in three parasitic anisakid nematodes. Oikos. 2007;116(10):1737–47. doi: 10.1111/j.0030-1299.2007.16016.x

[56] Valles-Vega I, Molina-Fernandez D, Benitez R, Hernandez-Trujillo S, Adroher FJ. Early development and life cycle of Contracaecum multipapillatum s.l. from a brown pelican Pelecanus occidentalis in the Gulf of California, Mexico. Dis Aquat Organ. 2017;125(3):167–78. doi: 10.3354/dao03147 . 28792415

[57] Mladineo I, Charouli A, Jelic F, Chakroborty A, Hrabar J. In vitro culture of the zoonotic nematode Anisakis pegreffii (Nematoda, Anisakidae). Parasit Vectors. 2023;16(1):51. Epub 20230202. doi: 10.1186/s13071-022-05629-5 ; PubMed Central PMCID: PMC9896804. 36732837 PMC9896804

[58] Angeles-Hernandez JC, Gomez-de Anda FR, Reyes-Rodriguez NE, Vega-Sanchez V, Garcia-Reyna PB, Campos-Montiel RG, et al. Genera and Species of the Anisakidae Family and Their Geographical Distribution. Animals (Basel). 2020;10(12). Epub 20201211. doi: 10.3390/ani10122374 ; PubMed Central PMCID: PMC7763134. 33322260 PMC7763134

[59] Li M, Wang C, Wang J, Li A, Gong X, Ma H. Redescription of Balantidium polyvacuolum Li 1963 (Class: Litostomatea) inhabiting the intestines of Xenocyprinae fishes in Hubei, China. Parasitol Res. 2009;106(1):177–82. Epub 20091029. doi: 10.1007/s00436-009-1645-0 . 19865830

[60] Feng S. Studies on a new ciliate, Balantidium fulinensis sp. nov., from the intestine of fishes. 1992.

[61] Hajipour N, Valizadeh H, Ketzis J. A review on fish-borne zoonotic parasites in Iran. Vet Med Sci. 2023;9(2):748–77. Epub 20221021. doi: 10.1002/vms3.981 ; PubMed Central PMCID: PMC10029912. 36271486 PMC10029912

[62] Hermosilla C, Hirzmann J, Silva LMR, Scheufen S, Prenger-Berninghoff E, Ewers C, et al. Gastrointestinal Parasites and Bacteria in Free-Living South American Sea Lions (Otaria flavescens) in Chilean Comau Fjord and New Host Record of a Diphyllobothrium scoticum-Like Cestode. Frontiers in Marine Science. 2018;5. doi: 10.3389/fmars.2018.00459

[63] Zhao W, Bu X, Zhou W, Zeng Q, Qin T, Wu S, et al. Interactions between Balantidium ctenopharyngodoni and microbiota reveal its low pathogenicity in the hindgut of grass carp. BMC Microbiol. 2024;24(1):7. Epub 20240103. doi: 10.1186/s12866-023-03154-8 ; PubMed Central PMCID: PMC10762984. 38172646 PMC10762984

[64] Martins ML, Cardoso L, Marchiori N, Benites de Padua S. Protozoan infections in farmed fish from Brazil: diagnosis and pathogenesis. Rev Bras Parasitol Vet. 2015;24(1):1–20. doi: 10.1590/S1984-29612015013 . 25909248

[65] Wudarski J, Simanov D, Ustyantsev K, de Mulder K, Grelling M, Grudniewska M, et al. Efficient transgenesis and annotated genome sequence of the regenerative flatworm model Macrostomum lignano. Nat Commun. 2017;8(1):2120. Epub 20171214. doi: 10.1038/s41467-017-02214-8 ; PubMed Central PMCID: PMC5730564. 29242515 PMC5730564

[66] Ladurner P, Scharer L, Salvenmoser W, Rieger RM. A new model organism among the lower Bilateria and the use of digital microscopy in taxonomy of meiobenthic Platyhelminthes: Macrostomum lignano, n. sp. (Rhabditophora, Macrostomorpha). Journal of Zoological Systematics and Evolutionary Research. 2005;43(2):114–26. doi: 10.1111/j.1439-0469.2005.00299.x

[67] Diez YL, Sanjuan C, Bosch C, Catalá A, Monnens M, Curini-Galletti M, et al. Diversity of free-living flatworms (Platyhelminthes) in Cuba. Biological Journal of the Linnean Society. 2023;140(3):423–33. doi: 10.1093/biolinnean/blad041

[68] Lee JJ, Jung BK, Lim H, Lee MY, Choi SY, Shin EH, et al. Comparative morphology of minute intestinal fluke eggs that can occur in human stools in the republic of Korea. Korean J Parasitol. 2012;50(3):207–13. Epub 20120813. doi: 10.3347/kjp.2012.50.3.207 ; PubMed Central PMCID: PMC3428565. 22949747 PMC3428565

[69] Chai JY, Jung BK. Fishborne zoonotic heterophyid infections: An update. Food Waterborne Parasitol. 2017;8–9:33–63. Epub 20170908. doi: 10.1016/j.fawpar.2017.09.001 ; PubMed Central PMCID: PMC7034020. 32095640 PMC7034020

[70] Phillips G, Hudson DM, Chaparro-Gutiérrez JJ. Presence of Paragonimus species within secondary crustacean hosts in Bogotá, Colombia. Revista Colombiana de Ciencias Pecuarias. 2019;32(2):150–7. doi: 10.17533/udea.rccp.v32n2a08

[71] Velez ID, Ortega J, Hurtado MI, Salazar AL, Robledo SM, Jimenez JN, et al. Epidemiology of paragonimiasis in Colombia. Trans R Soc Trop Med Hyg. 2000;94(6):661–3. doi: 10.1016/s0035-9203(00)90223-2 . 11198651

[72] Donato RA, Donato RJ. Pulmonary, liver and cerebral paragonimiasis: An unusual clinical case in Colombia. Travel Med Infect Dis. 2022;46:102253. Epub 20211230. doi: 10.1016/j.tmaid.2021.102253 . 34974180

[73] Fiksdal L, Pommepuy M, Caprais MP, Midttun I. Monitoring of fecal pollution in coastal waters by use of rapid enzymatic techniques. Appl Environ Microbiol. 1994;60(5):1581–4. doi: 10.1128/aem.60.5.1581-1584.1994 ; PubMed Central PMCID: PMC201520. 8017937 PMC201520

[74] Manini E, Baldrighi E, Ricci F, Grilli F, Giovannelli D, Intoccia M, et al. Assessment of Spatio-Temporal Variability of Faecal Pollution along Coastal Waters during and after Rainfall Events. Water. 2022;14(3). doi: 10.3390/w14030502

[75] Li E, Saleem F, Edge TA, Schellhorn HE. Biological Indicators for Fecal Pollution Detection and Source Tracking: A Review. Processes. 2021;9(11). doi: 10.3390/pr9112058

[76] Procop GW. North American paragonimiasis (Caused by Paragonimus kellicotti) in the context of global paragonimiasis. Clin Microbiol Rev. 2009;22(3):415–46. doi: 10.1128/CMR.00005-08 ; PubMed Central PMCID: PMC2708389. 19597007 PMC2708389

[77] Saraiva A, Eiras JC, Cruz C, Xavier R. Synopsis of the Species of Coccidians Reported in Marine Fish. Animals (Basel). 2023;13(13). Epub 20230626. doi: 10.3390/ani13132119 ; PubMed Central PMCID: PMC10339986. 37443917 PMC10339986

[78] Whipps CM, Fournie JW, Morrison DA, Azevedo C, Matos E, Thebo P, et al. Phylogeny of fish-infecting Calyptospora species (Apicomplexa: Eimeriorina). Parasitol Res. 2012;111(3):1331–42. Epub 20120529. doi: 10.1007/s00436-012-2969-8 . 22645034

[79] Arredondo NJ, Gil de Pertierra AA. Pseudoacanthocephalus lutzi (Hamann, 1891) comb. n. (Acanthocephala: Echinorhynchidae) for Acanthocephalus lutzi (Hamann, 1891), parasite of South American amphibians. Folia Parasitol (Praha). 2009;56(4):295–304. doi: 10.14411/fp.2009.034 . 20128242

[80] Drago FB. Macroparásitos. Diversidad y Biología: Editorial de la Universidad Nacional de La Plata (EDULP); 2017.

[81] Piekarski G, Mehlhorn H. Fundamentos de parasitología. Parásitos del hombre y de los animales domésticos. 1993.

[82] Özcan M, Yilmaz Y, Donat E, Kılavuz D, Tuncel M. A Research on Endoparasitic Fauna in Fish Species Caught in Menzelet Dam Lake KahramanmaraŞ (Turkey). Middle East Journal of Science. 2019;5(1):33–40. doi: 10.23884/mejs.2019.5.1.04

[83] Amin OM, Rubtsova NY, Ha NV. Description of Three New Species of Rhadinorhynchus Luhe, 1911 (Acanthocephala: Rhadinorhynchidae) from Marine Fish off the Pacific Coast of Vietnam. Acta Parasitol. 2019;64(3):528–43. Epub 20190703. doi: 10.2478/s11686-019-00092-2 . 31270659

[84] Salgado-Maldonado G, Novelo-Turcotte MT. Acanthocephalus amini n. sp. (Acanthocephala: Echinorhynchidae) from the freshwater fish Cichlasoma urophthalmus (Gunther) (Cichlidae) in Mexico. Syst Parasitol. 2009;73(3):193–8. Epub 20090527. doi: 10.1007/s11230-009-9189-3 . 19472078

[85] Tantaleán M, Sánchez L, Gómez L, Huiza A. Acantocéfalos del Perú. Revista Peruana de Biología. 2005;12:83–92.

[86] Santos C, Gibson D, Tavares L, Ler, Luque J. Checklist Of Acanthocephala Associated With The Fishes Of Brazil. Zootaxa 1938:. 2008;1938:22. doi: 10.5281/zenodo.184999

[87] Olivera LA, Campiao KM. Diversity of Acanthocephala parasites in Neotropical amphibians. J Helminthol. 2024;98:e11. Epub 20240124. doi: 10.1017/S0022149X23000986 . 38263742

[88] Lozano-Duque Y, Vidal LA, S.3 GRN. Listado de diatomeas (Bacillariophyta) registradas para el Mar Caribe colombiano. Bol Invest Mar Cost. 2010;39(1). doi: http://hdl.handle.net/1834/3741.

[89] Hoyos Acuña JJ, Quintana-Manotas HL, Bermudez Rivas C, Molina Triana AF, Castrilllón Valencia FA, Parada Gutiérrez JL. Listado de especies de fitoplancton en la bahía de Tumaco, Pacífico colombiano. Intropica. 2021:214–31. doi: 10.21676/23897864.4064

[90] Cardoso JSO. Comunidad fitoplanctonica de tres áreas portuarias del Pacifico Colombiano y su relación con algunas variables ambientales, inclusive el trafico marino Jorge Tadeo Lozano; 2019.

[91] Mohan R, Shanvas S, Thamban M, Sudhakar M. Spatial distribution of diatoms in surface sediments from the Indian sector of Southern Ocean. Current Science. 2006;91:1495–502.

[92] Van Den Hoek C, Mann D, Johns H. Algae An Introduction to Phycology. Cambridge University Press; Cambridge, UK: 1997.

[93] Scholz B, Guillou L, Marano AV, Neuhauser S, Sullivan BK, Karsten U, et al. Zoosporic parasites infecting marine diatoms ‐ A black box that needs to be opened. Fungal Ecol. 2016;19:59–76. doi: 10.1016/j.funeco.2015.09.002 ; PubMed Central PMCID: PMC5221735. 28083074 PMC5221735

[94] Ferrazzano GF, Papa C, Pollio A, Ingenito A, Sangianantoni G, Cantile T. Cyanobacteria and Microalgae as Sources of Functional Foods to Improve Human General and Oral Health. Molecules. 2020;25(21). Epub 20201106. doi: 10.3390/molecules25215164 ; PubMed Central PMCID: PMC7664199. 33171936 PMC7664199

[95] Paasche E. A review of the coccolithophorid Emiliania huxleyi (Prymnesiophyceae), with particular reference to growth, coccolith formation, and calcification-photosynthesis interactions. Phycologia. 2002;41(5):503–29.

[96] Lepage G, Roy CC. Improved recovery of a fatty acid from silage extracts with chloroform-methanol. Journal of Lipid Research. 1988;29(11):1579–83.

[97] Pohnert G. Diatom/copepod interactions in plankton: the indirect chemical defense of unicellular algae. Chemical Ecology. 2000;14(5): 895–901. 10.1002/cbic.200400348 15883976

[98] De Souza MP, Rivera ING, Jett M. Identification and characterization of two anionic antimicrobial peptides from the marine diatom, Cylindrotheca fusiformis. The Journal of Peptide Research. 1999;53(6):640–9.

[99] Wiewióra M. Diatomaceous Earth in the Prevention of Worm Infestation in Purebred Pigeons. Annals of Warsaw University of Life Sciences- SGGW Animal Science. 2015;(2):161–6.

[100] Ikusika OO, Mpendulo CT, Zindove TJ, Okoh AI. Fossil Shell Flour in Livestock Production: A Review. Animals (Basel). 2019;9(3). Epub 20190226. doi: 10.3390/ani9030070 ; PubMed Central PMCID: PMC6466221. 30813550 PMC6466221

[101] Soji-Mbongo Z, Mpendulo TC. Knowledge Gaps on the Utilization of Fossil Shell Flour in Beef Production: A Review. Animals (Basel). 2024;14(2). Epub 20240121. doi: 10.3390/ani14020333 ; PubMed Central PMCID: PMC10812526. 38275794 PMC10812526

[102] Moran MC, Magaña FG. Dieta y hábitos alimenticios de la corvina amarilla Cynoscion albus en el Pacífico ecuatoriano. La Técnica. 2017. 1390–6895.

[103] Selvaraj JJ, Rosero-Henao LV, Cifuentes-Ossa MA. Projecting future changes in distributions of small-scale pelagic fisheries of the southern Colombian Pacific Ocean. Heliyon. 2022;8(2):e08975. Epub 20220218. doi: 10.1016/j.heliyon.2022.e08975 ; PubMed Central PMCID: PMC8866063. 35243094 PMC8866063

[104] Calle-Morán MD, Corrales-Moreira JM, Quimís-Calle AV. Size structure, length-body mass relationship and condition factor of three corvinas species (Sciaenidae) in the Ecuadorian Pacific Ocean. 2022. doi: 10.54140/raop.v4i1.54

[105] Salgado NAN, Fernández GE, Rodríguez VD, Díaz EG, Crespo LP, Gordillo PR, et al. Análisis del contenido estomacal de la lisa (Mugil curema Cuvier y Valencinnes) en Playa Navarro, Vega de Alatorre, Veracruz. Revista de Zoología. 2017;28:9–18.

[106] Rueda PSn. Stomach content analysis of Mugil cephalus and Mugil curema (Mugiliformes: Mugilidae) with emphasis on diatoms in the Tamiahua lagoon, México. Rev Biol Trop. 2002;50(1). doi: https://hdl.handle.net/10669/26603. 12298252

[107] Nikolic N, Morandeau G, Hoarau L, West W, Arrizabalaga H, Hoyle S, et al. Review of albacore tuna, Thunnus alalunga, biology, fisheries and management. Reviews in Fish Biology and Fisheries. 2016;27(4):775–810. doi: 10.1007/s11160-016-9453-y

[108] Mondal S, Lee M-A. Habitat modeling of mature albacore (Thunnus alalunga) tuna in the Indian Ocean. Frontiers in Marine Science. 2023;10. doi: 10.3389/fmars.2023.1258535

[109] Williams AJ, Allain V, Nicol SJ, Evans KJ, Hoyle SD, Dupoux C, et al. Vertical behavior and diet of albacore tuna (Thunnus alalunga) vary with latitude in the South Pacific Ocean. Deep Sea Research Part II: Topical Studies in Oceanography. 2015;113:154–69. doi: 10.1016/j.dsr2.2014.03.010

[110] G da Silva Cu. A checklist of metazoan parasites from albacore Thunnus alalunga (Bonaterre, 1788). Journal of Aquaculture & Marine Biology. 2018;7(1):52–3. doi: 10.15406/jamb.2018.07.00183.

[111] Mele S, Merella P, Macias D, Gómez MJ, Garippa G, Alemany F. Metazoan gill parasites of wild albacore Thunnus alalunga (Bonaterre, 1788) from the Balearic Sea (western Mediterranean) and their use as biological tags. Fisheries Research. 2010;102(3):305–10. doi: 10.1016/j.fishres.2010.01.002

[112] Eslami A, Sabokroo H, Sh. R-B. Infection of Anisakids Larvae in Long Tail Tuna (Thunnus tonggol) In North Persian Gulf. Iran J Parasitol. 2011;6(3):96–100. PubMed Central PMCID: PMC3279884. 22347303 PMC3279884

[113] Knoff M, Fonseca M, Nunes N, Dos Santos A, Clemente S, Kohn A, et al. Anisakidae and Raphidascarididae nematodes of tuna (Perciformes: Scombridae) from state of Rio de Janeiro, Brazil. Neotropical Helminthology. 2017;11:45–52.

[114] Villalba-Vasquez PJ, Violante-Gonzalez J, Pulido-Flores G, Monks S, Rojas-Herrera AA, Flores-Rodriguez P, et al. Parasite communities of the spotted rose snapper Lutjanus guttatus (Perciformes: Lutjanidae) off the Mexican Pacific coasts: Spatial and long-term inter-annual variations. Parasitol Int. 2022;88:102551. Epub 20220131. doi: 10.1016/j.parint.2022.102551 . 35101604

[115] Mar-Silva AF, Diaz-Jaimes P, Dominguez-Mendoza C, Dominguez-Dominguez O, Valdiviezo-Rivera J, Espinoza-Herrera E. Genomic assessment reveals signal of adaptive selection in populations of the Spotted rose snapper Lutjanus guttatus from the Tropical Eastern Pacific. PeerJ. 2023;11:e15029. Epub 20230327. doi: 10.7717/peerj.15029 ; PubMed Central PMCID: PMC10062342. 37009151 PMC10062342

[116] Correa-Herrera T, Jiménez-Segura LF. [Reproductive biology of Lutjanus guttatus (Perciformes: Lutjanidae) in Utria National Park, Colombian Pacific]. Rev Biol Trop. 2013;61(2):829–40. . 23885593

[117] Vergara-Flórez V, Consuegra A. Contracaecum sp. (Nematode: Anisakidae) en peces de interés comercial en el golfo de Morrosquillo, Sucre ‐ Colombia. Gestión y Ambiente. 2021;24(2). doi: 10.15446/ga.v24n2.97356

[118] Administration UFaD. Fish and fisheries products hazards and controls guidance. Available at http://www.fda.gov/Food/GuidanceComplianceRegulatoryInformation/GuidanceDocuments/Seafood/FishandFisheriesProductsHazardsandControlsGuide/defaulthtm, 2011. Available from: https://www.fda.gov/food/seafood-guidance-documents-regulatory-information/fish-and-fishery-products-hazards-and-controls.

[119] Lõhmus M, Björklund M. Climate change: what will it do to fish-parasite interactions? Biological Journal of the Linnean Society. 2015;116(2):397–411. doi: 10.1111/bij.12584

