# Supplementary material for: Diatoms-endoparasite association in fish from the marine pacific coast of Colombia (Buenaventura)
Source: PLoS One. 2024 Dec 27;19(12):e0312015. doi: 10.1371/journal.pone.0312015 (PMC11676577; doi:10.1371/journal.pone.0312015)
Supplement: S1 Fig — Notes the different species of diatoms found in the fish species collected from Buenaventura harbor -Colombia. (DOCX) [file pone.0312015.s001.docx]

S1 Fig**.** First, second and third sampling. Notes the different species of diatoms found in the fish species collected from Buenaventura harbor -Colombia.

**
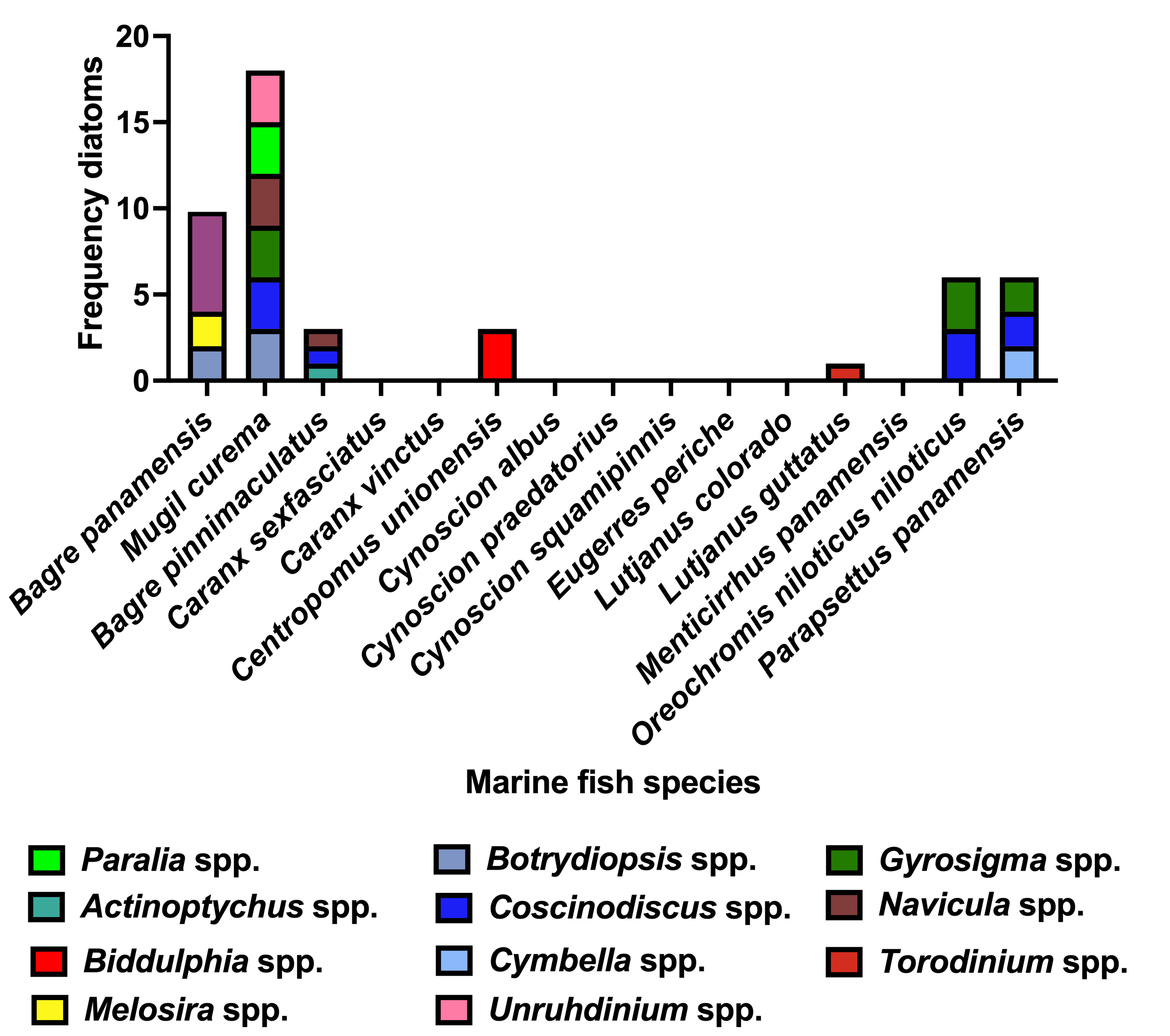
**

**S1.** First sampling March 19 2023. Notes the different species of diatoms found in the fish species collected from Bunaventura harbor -Colombia.


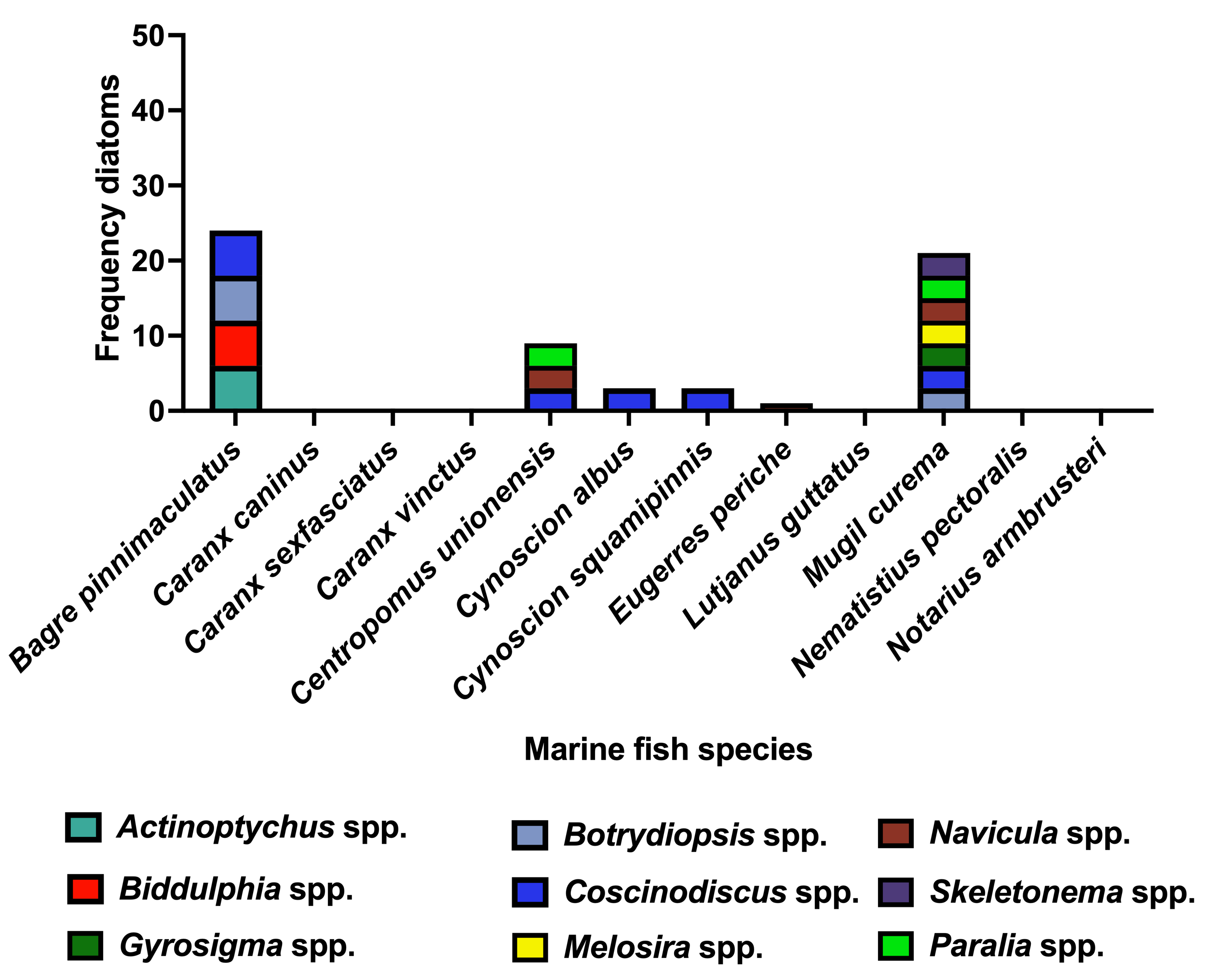


**S1.** Second sampling Oct 07 2023. Notes the different species of diatoms found in the fish species collected from Buenaventura harbor -Colombia.


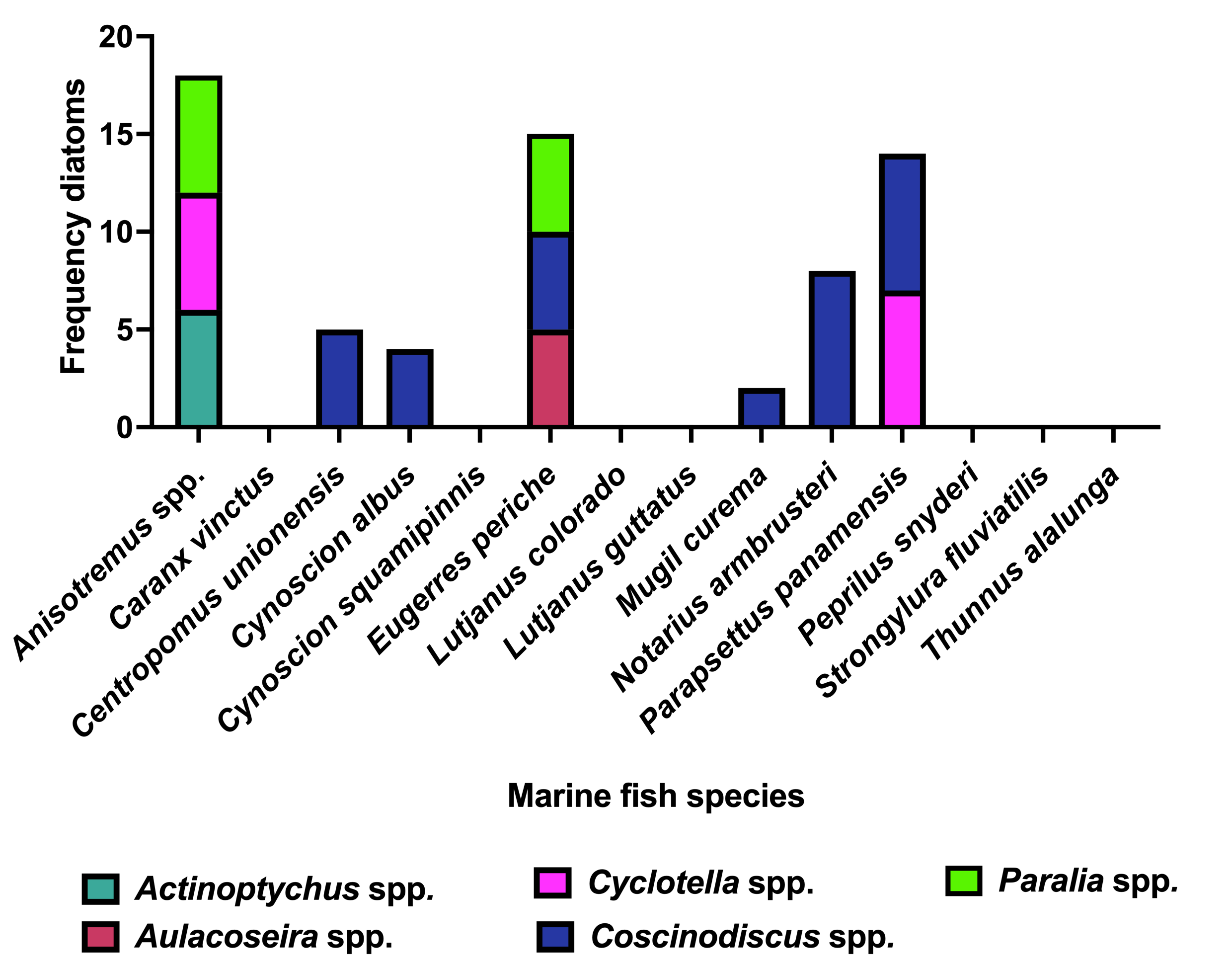


**S1.** Third sampling March 09 2024. Notes the different species of diatoms found in the fish species collected from Buenaventura harbor -Colombia.
