# Supplementary material for: Diatoms-endoparasite association in fish from the marine pacific coast of Colombia (Buenaventura)
Source: PLoS One. 2024 Dec 27;19(12):e0312015. doi: 10.1371/journal.pone.0312015 (PMC11676577; doi:10.1371/journal.pone.0312015)
Supplement: S2 Fig — Association of parasites and diatoms positive in fish sampling. (DOCX) [file pone.0312015.s002.docx]

**S3 Fig.** Different time sampling. Association of parasites and diatoms positive in fish sampling.


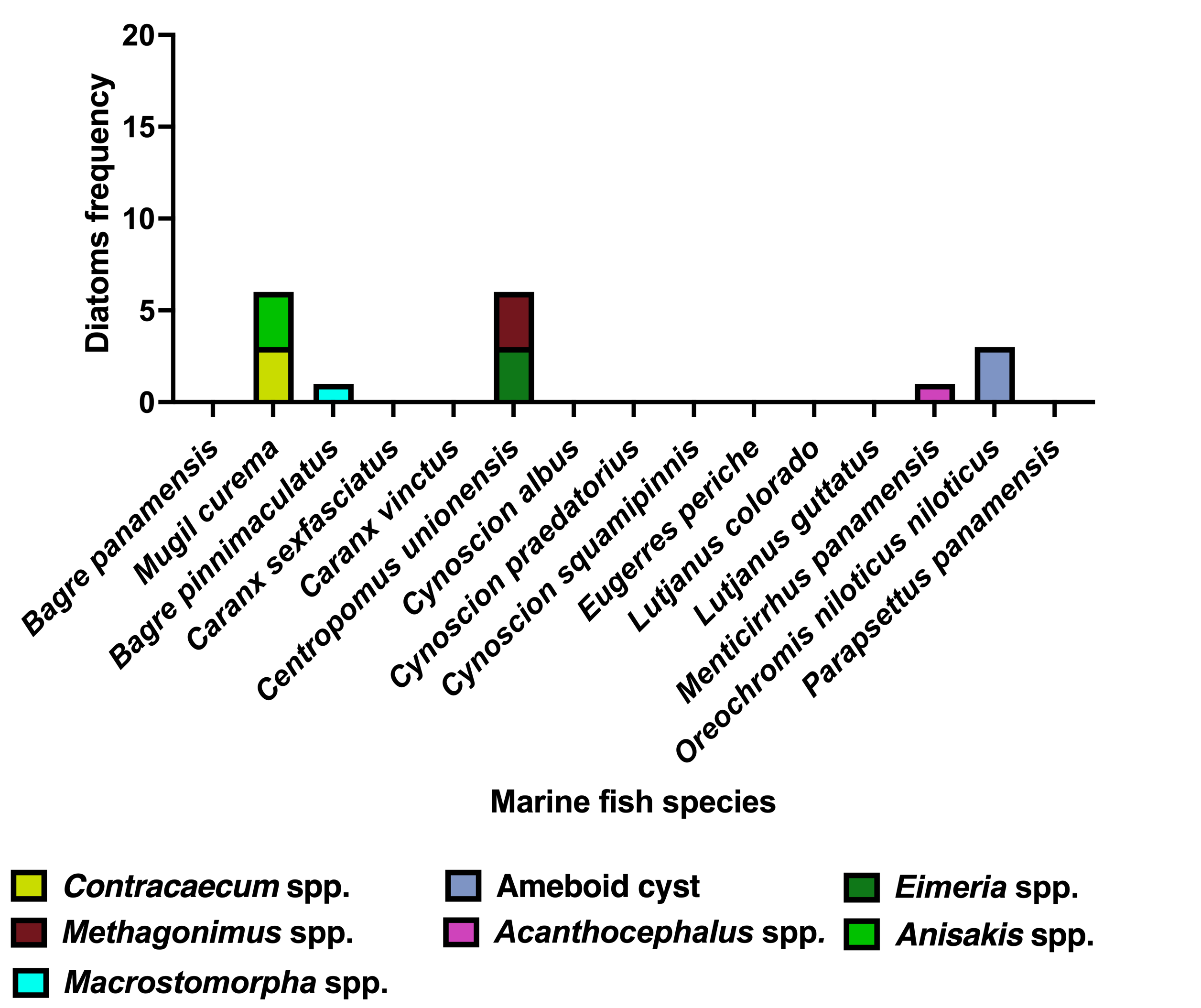


**S3. Fig 1.** **First sampling March 19 2023.** Association of parasites and diatoms positive in fish sampling.

**S3. Fig 2.** **Second sampling Oct 07 2023.** Association of parasites and diatoms positive in fish sampling.


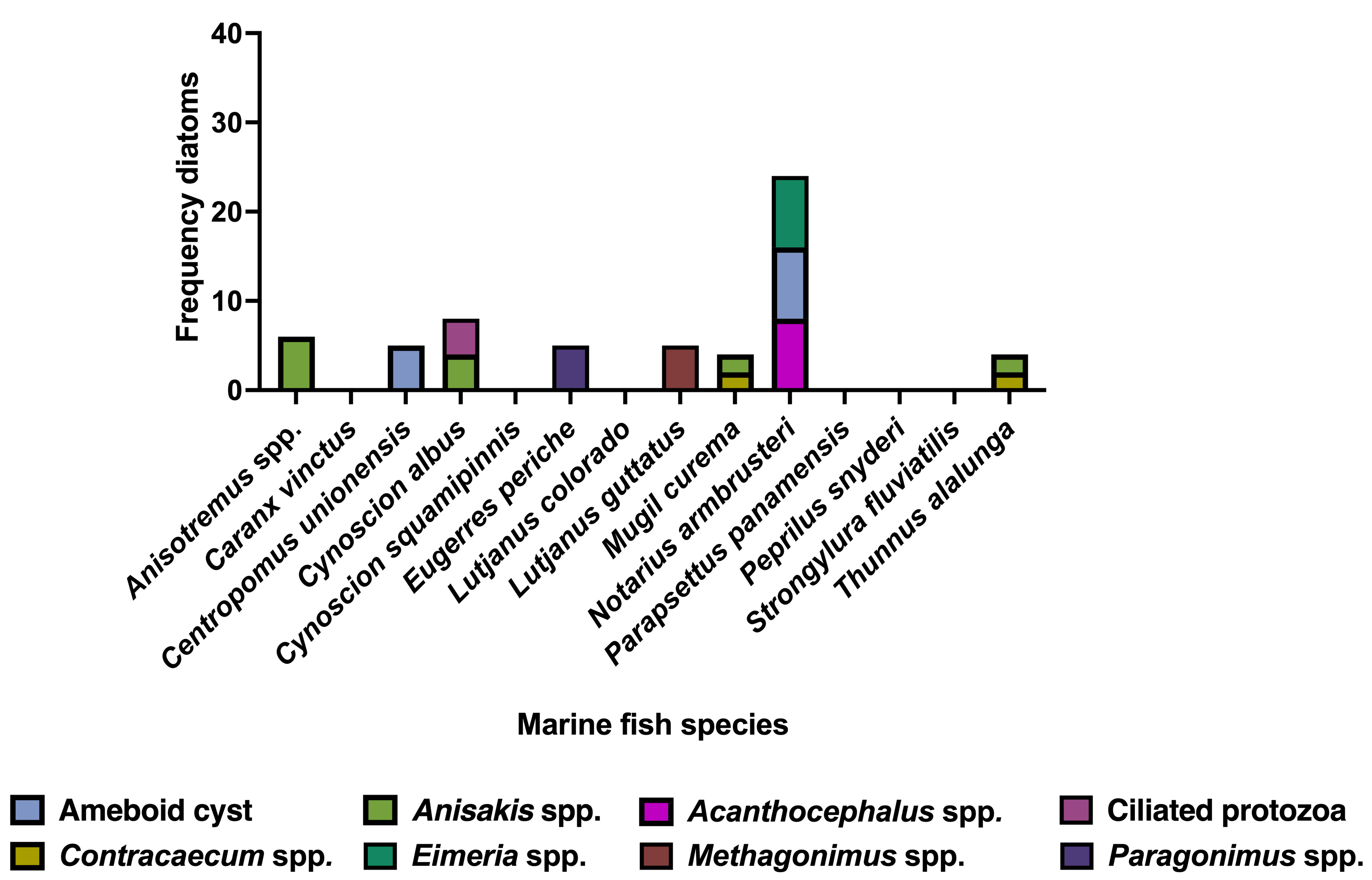


**S3. Fig 3.** **Third sampling March 09 2024.** Association of parasites and diatoms positive in fish sampling.

**S3.** **Fig 4.** Total prevalence of parasites in fish from harbor of Buenaventura – Colombia. P ≤0.0025****.** P≤0.0002*******

**
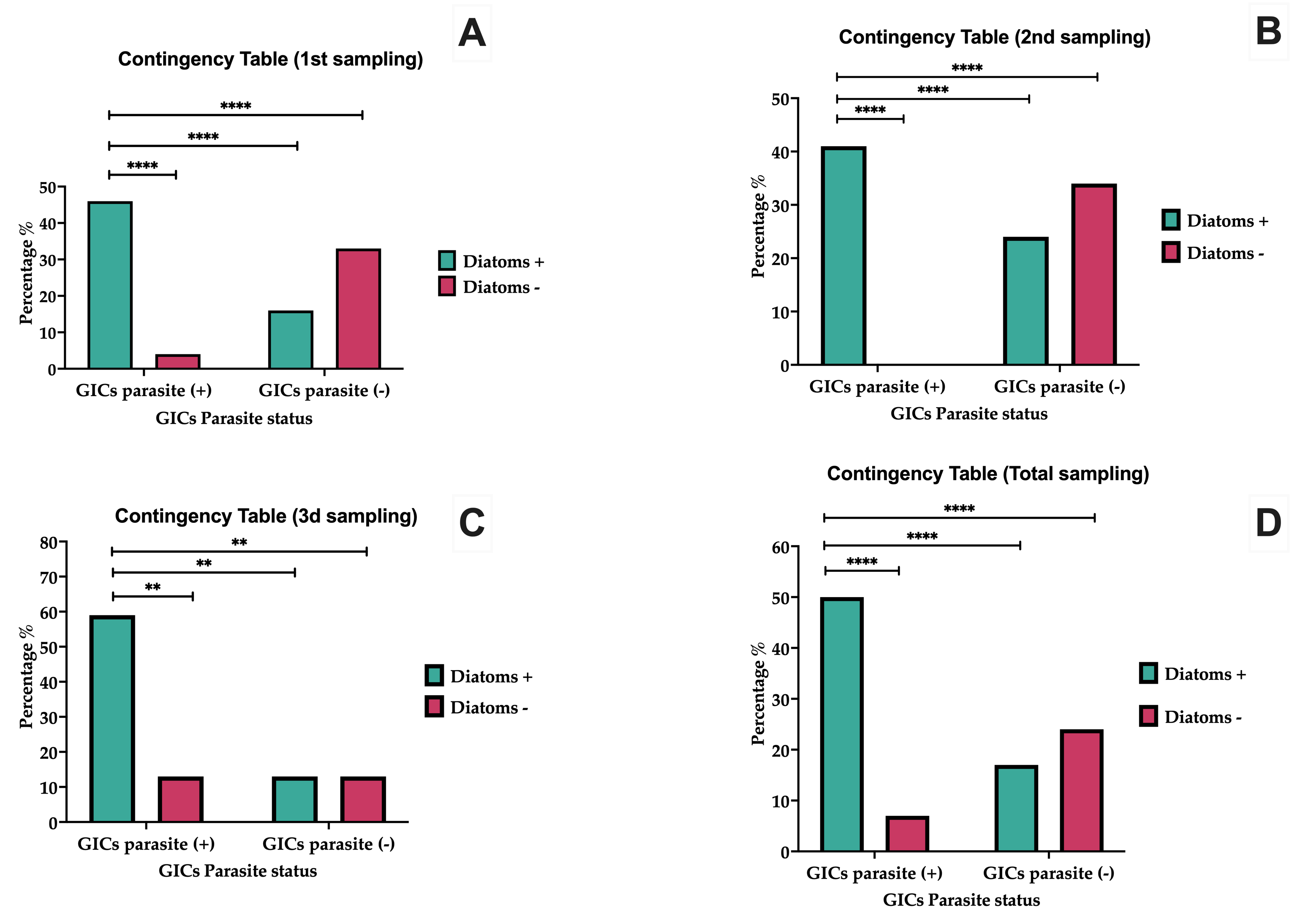
**

**S3. Fig 5. Contingency table.**  Parasites - Diatoms assocition in Gastrointestinal Compartments (CGIs) in fish from harbor of Buenaventura – Colombia. First sampling 19 march 2023 (A), Second sampling 07 october 2023 (B), Third sampling 09 march 2024 (C), Total sampling (D). Significant differences using Fisher test P ≤0,0035, ****.** P≤0.0001********
