# Supplementary material for: Diatoms-endoparasite association in fish from the marine pacific coast of Colombia (Buenaventura)
Source: PLoS One. 2024 Dec 27;19(12):e0312015. doi: 10.1371/journal.pone.0312015 (PMC11676577; doi:10.1371/journal.pone.0312015)
Supplement: S1 Table — (DOCX) [file pone.0312015.s003.docx]

S1 Table**.** Diatoms and parasites are found in the gastrointestinal compartments of the fish population in Buenaventura port of Colombia.

| **1st sampling. March 19^th^-2023.** | | | | | | |
| --- | --- | --- | --- | --- | --- | --- |
| **Fish specie** | **N0. Digestive compartments** | | **Diatoms** | | **Prevalence %** | **Parasite association** |
| *Bagre panamensis* | *2* | | *Botrydiopsis* spp. | | 8.3 | Negative |
|  |  |  | *Melosira* spp. | | 8.3 | Negative |
| *Mugil curema* | 3 | | *Botrydiopsis* spp. | | 12.5 | *Contracaecum* spp. *Anisakis* spp. |
|  |  |  | *Coscinodiscus* spp. | | 12.5 |  |
|  |  |  | Gyrosigma spp. | | 12.5 |  |
|  |  |  | *Navicula* spp. | | 12.5 |  |
|  |  |  | *Paralia* spp. | | 12.5 |  |
|  |  |  | *Unruhdinium* spp. | | 12.5 |  |
| *Bagre pinnimaculatus* | 1 | | *Actinoptychus* spp. | | 4.2 | *Macrostomorpha* spp. |
|  |  |  | *Coscinodiscus* spp. | | 4.2 |  |
|  |  |  | *Navicula* spp. | | 4.2 |  |
| *Caranx sexfasciatus* | 1 | | Negative | | 0 | Negative |
| *Caranx vinctus* | 1 | | Negative | | 0 | Negative |
| *Centropomus unionensis* | 3 | | *Biddulphia* spp. | | 12.5 | *Eimeria* spp., *Methagonimus* spp. |
| *Cynoscion albus* | 2 | | Negative | | 0 | Negative |
| *Cynoscion praedatorius* | 1 | | Negative | | 0 | Negative |
| *Cynoscion squamipinnis* | 1 | | Negative | | 0 | Negative |
| *Eugerres periche* | 1 | | Negative | | 0 | Negative |
| *Lutjanus colorado* | 1 | | Negative | | 0 | Negative |
| *Lutjanus guttatus* | 1 | | *Torodinium* spp. | | 0 | Negative |
| *Menticirrhus panamensis* | 1 | | Negative | | 0 | *Acanthocephalus* spp. |
| *O. niloticus niloticus* | 3 | | *Coscinodiscus* spp. | | 12.5 | Ameboid cyst |
|  |  |  | *Gyrosigma* spp. | | 12.5 | Ameboid cyst |
| *Parapsettus panamensis* | 2 | | *Coscinodiscus* spp. | | 8.3 | Negative |
|  |  |  | *Cymbella* spp*.* | | 8.3 | Negative |
|  |  |  | *Gyrosigma* spp. | | 8.3 | Negative |
| **2nd. Sampling. October 7^th^-2023.** | | | | | | |
| **Fish species** | | **Digestive compartments** | **Diatoms** | | **Prevalence %** | **Parasite association** |
| *Bagre pinnimaculatus* | | *6* | *Aulacoseira* spp. | | 20.7 | *Anisakis* spp.  Ameboid cyst |
|  |  |  | *Botrydiopsis* spp*.* | | 20.7 |  |
|  |  |  | *Coscinodiscus* spp*.* | | 20.7 |  |
|  |  |  | *Cyclotella* spp*.* | | 20.7 |  |
| *Caranx caninus* | | 3 | Negative | | 0 | Negative |
| *Caranx sexfasciatus* | | 2 | Negative | | 0 | Negative |
| *Caranx vinctus* | | 1 | Negative | | 0 | Negative |
| *Centropomus unionensis* | | *3* | *Coscinodiscus* spp*.* | | 10.3 | Negative |
|  |  |  | *Navicula* spp*.* | | 10.3 |  |
|  |  |  | *Paralia* spp*.* | | 10.3 |  |
| *Cynoscion albus* | | *3* | *Coscinodiscus* spp. | | 10.3 | *Anisakis* spp. *Balantidium* spp. |
| *Cynoscion squamipinnis* | | 3 | *Coscinodiscus* spp. | | 10.3 | Negative |
| *Eugerres periche* | | 1 | *Navicula* spp*.* | | 3.4 | Negative |
| *Lutjanus guttatus* | | *2* | Negative | | 0 | Negative |
| *Mugil curema* | | *3* | *Coscinodiscus* spp*.* | | 10.3 | *Anisakis* spp.  *Contracaecum* spp.  Ameboid cyst |
|  |  |  | *Cyclotella* spp*.* | | 10.3 |  |
|  |  |  | *Gyrosigma* spp*.* | | 10.3 |  |
|  |  |  | *Melosira* spp. | | 10.3 |  |
|  |  |  | *Navicula* spp*.* | | 10.3 |  |
|  |  |  | *Paralia* spp*.* | | 10.3 |  |
|  |  |  | *Skeletonema* spp*.* | | 10.3 |  |
| *Nematistius pectoralis* | | 1 | Negative | | 0 | Negative |
| *Notarius armbrusteri* | | 1 | Negative | | 0 | Negative |
| **3rd. Sampling March 9^th^-2024.** | | | | | | |
| **Fish specie** | | **Total digestive compartments (fish species)** | | **Diatoms** | **Prevalence %** | **Parasite association** |
| *Anisotremus spp* | | 6 | | *Actynoptychus* spp*.* | 11.8 | *Anisakis* spp. |
|  |  |  |  | *Cyclotella* spp*.* | 11.8 |  |
|  |  |  |  | *Paralia* spp*.* | 11.8 |  |
| *Caranx vinctus* | | 1 | | Negative | 2 | Negative |
| *Centropomus unionensis* | | 5 | | *Coscinodiscus* spp. | 9.8 | Ameboid cys |
| *Cynoscion albus* | | 4 | | *Coscinodiscus* spp. | 7.8 | *Anisakis* spp.  *Balantidium* spp. |
| *Cynoscion squamipinnis* | | 1 | | Negative | 0 | Negative |
| *Eugerres periche* | | 5 | | *Aulacoseira* spp*.* | 9.8 | *Paragonimus* spp. |
|  |  |  |  | *Coscinodiscus* spp*.* | 9.8 |  |
|  |  |  |  | *Paralia* spp*.* | 9.8 |  |
| *Lutjanus colorado* | | 3 | | Negative | 0 | Negative |
| *Lutjanus guttatus* | | 5 | | Negative | 0 | *Metagonimus* spp. |
| *Mugil curema* | | 2 | | *Coscinodiscus* spp. | 3.9 | *Anisakis* spp.  *Contracaecum* spp. |
| *Notarius armbrusteri* | | 8 | | *Coscinodiscus* spp. | 15.7 | *Acanthocephalus* spp.  Eimeria spp.  Ameboid cys |
| *Parapsettus panamensis* | | 7 | | *Coscinodiscus* spp. | 13.7 | Negative |
|  |  |  |  | *Cyclotella* spp. | 2 | Negative |
| *Peprilus snyderi* | | 1 | | Negative | 0 | Negative |
| *Strongylura fluviatilis* | | 1 | | Negative | 0 | Negative |
| *Thunnus alalunga* | | 2 | | Negative | 0 | *Anisakis* spp.  *Contracaecum* spp. |
